# Cytotoxic mixed-ligand complexes of Cu(II): A combined experimental and computational study

**DOI:** 10.3389/fchem.2022.1028957

**Published:** 2022-09-29

**Authors:** Mamaru Bitew Alem, Tadewos Damena, Tegene Desalegn, Moses Koobotse, Rajalakshmanan Eswaramoorthy, Kennedy J. Ngwira, Japheth O. Ombito, Matshediso Zachariah, Taye B. Demissie

**Affiliations:** ^1^ Department of Applied Chemistry, Adama Science and Technology University, Adama, Ethiopia; ^2^ School of Allied Health Professions, University of Botswana, Gaborone, Botswana; ^3^ Department of Biomaterials, Saveetha Dental College and Hospitals, Saveetha Institute of Medical and Technical Sciences, Saveetha University, Chennai, India; ^4^ Molecular Sciences Institute, School of Chemistry, University of the Witwatersrand, Johannesburg, South Africa; ^5^ Department of Chemistry, University of Botswana, Gaborone, Botswana

**Keywords:** cytotoxicity, Cu(II) complexes, antibacterial activity, BOILED-egg, MCF-7, DFT, molecular docking

## Abstract

Herein, we report the synthesis of mixed-ligand Cu(II) complexes of metformin and ciprofloxacin drugs together with 1,10-phenanthroline as a co-ligand. The synthesized complexes were characterized using different spectroscopic and spectrometric techniques. *In vitro* cytotoxic activity against human breast adenocarcinoma cancer cell line (MCF-7) as well as antibacterial activity against two gram-negative and two gram-positive bacterial strains were also investigated. The analyses of the experimental results were supported using quantum chemical calculations and molecular docking studies against estrogen receptor alpha (ERα; PDB: 5GS4). The cytotoxicity of the [Cu(II) (metformin) (1,10-phenanthroline)] complex (**1**), with IC_50_ of 4.29 µM, and the [Cu(II) (ciprofloxacin) (1,10-phenanthroline)] complex (**2**), with IC_50_ of 7.58 µM, were found to be more effective than the referenced drug, cisplatin which has IC_50_ of 18.62 µM against MCF-7 cell line. The molecular docking analysis is also in good agreement with the experimental results, with binding affinities of –7.35, –8.76 and –6.32 kcal/mol, respectively, for complexes **1**, **2** and cisplatin against ERα. Moreover, complex **2** showed significant antibacterial activity against *E. coli* (inhibition diameter zone, IDZ, = 17.3 mm), *P. aeruginosa* (IDZ = 17.08 mm), and *S. pyogen* (IDZ = 17.33 mm), at 25 μg/ml compared to ciprofloxacin (IDZ = 20.0, 20.3, and 21.3 mm), respectively. Our BOILED-egg model indicated that the synthesized metal complexes have potentially minimal neurotoxicity than that of cisplatin.

## Introduction

Cancer is one of the leading causes of death worldwide, accounting for an estimated 10 million deaths in 2020 (Ferlay et al., 2021). In the same period, there were approximately 19.5 million new cancer cases and 685,000 deaths with female breast cancer being the most diagnosed cancer (2.26 million cases) (Ferlay et al., 2021; Nishimura and Acoba, 2022). Chemotherapy is among the extensively utilized treatment methods in the fight against breast cancer (Shahidian et al., 2020). The discovery of antiproliferative effects of cisplatin and other platinum-based chemotherapeutic drugs has inspired researchers to focus on research related to transition metal-based coordination compounds for their antitumor activities (Buchtik et al., 2012). Cisplatin, oxaliplatin, carboplatin, nedaplatin, heptaplatin, and lobaplatin are square planar platinum-based chemotherapeutic drugs approved for human cancer treatment worldwide (Mitra, 2016). However, their toxic adverse effects such as hepatotoxicity, myelosuppression, nausea, neuropathy, nephrotoxicity, ototoxicity, and acquired/inherent drug resistances due to repeated doses are the main concerns among the various drawbacks of platinum-based chemotherapeutic agents. To overcome the aforementioned drawbacks, many attempts have been made to prepare complexes with improved cytotoxic effects against cancer cell lines and decreased negative side effects as compared to the platinum-based chemotherapeutic agents (Buchtik et al., 2012). One of the possible approaches proposed to reduce metal-related toxicity is the use of essential transition metals as central atom in the coordination sphere of a metal therapeutic agent. Cu(II) complexes have been used as representatives of these essential-endogenous metals because copper is one of the most important trace elements involved in several enzymatic and protein functions in metabolism (Ruiz-Azuara and E Bravo-Gomez, 2010; Correia et al., 2015; Boodram et al., 2016; Nunes et al., 2020). Promising activities of copper-based coordination compounds for target-specific next-generation anticancer therapeutics have previously been reported (Azam et al., 2018). Cu(II) complexes of 2-phenyl-3-hydroxy-4(1*H*)-quinolinone (O,O donor) and other nitrogen-containing (N,N donor) ligands, such as 2,2′-bibyrdine and 1,10-phenanthroline derivatives, showed promising cytotoxicity against human breast cancer cell lines (MCF-7) (Buchtik et al., 2012; Johnstone et al., 2016), while 1,10-phenanthroline and 2,2′-bipyridine based Cu(II) complexes showed superior *in vitro* and *in-vivo* antitumor activity when compared to cisplatin (Ruiz-Azuara and E Bravo-Gomez, 2010; Correia et al., 2015; Nunes et al., 2020). Casiopeinas® are a group of 1,10-phenanthroline and 2,2′-bipyridine (bipy) based Cu(II) square planar complexes with N-N-O-O type coordination, which have demonstrated antiproliferative and antineoplastic activity *in vitro* and *in vivo* experiments (Bravo-Gomez et al., 2009; Reina et al., 2021). Inspired by the promising results of Casiopeinas®, we designed the synthesis of Cu(II) based mixed-ligand metal complexes of N-N-N-N and N-N-O-O donor type ligands with promising cytotoxic activity in *in vitro* tests.

## Experimental

### Materials and methods

All chemicals and regents used for this study were analytical grade and used without further purification. 1,10-phenanthroline monohydrate (BDH chemical Ltd., Poole, England), copper (II) acetate monohydrate [Cu(CO_2_CH_3_)_2_·H_2_O], triethylamine, NaHCO_3_, NaOH, and Mueller-Hinton agar were purchased from Loba Chemie PVT Ltd., Addis Ababa. Metformin hydrogen chloride (Met.HCl) and ciprofloxacin hydrogen chloride (Cip.HCl) were obtained from Cadila Pharmaceuticals PLC, Ethiopia. Methanol, ethanol, HCl, DMSO, ethyl acetate and dichloromethane (DCM) were purchased from Alpha Chemika, Addis Ababa, Ethiopia. Thin Layer Chromatography (MERCK Silica gel 60 F254) together with UV Cabinet (UV-Vis lamp at 254 and 365 nm) were used to monitor the progress of the chemical reactions. The melting points of the complexes were determined using capillary tubes (Thiele tube). UV-Vis spectrophotometer (SM-1600 Spectrophotometer), FTIR (Perkin-Elmer BX spectrometer, Shimadzu Corporation, Japan), TGA/DTC (DTG-60H SHIMADZU thermal analyzer) and High resolution mass spectra (Waters-LCT-Premier mass spectrometer) were used to characterize the synthesized metal complexes.

### Synthesis of Cu(II) mixed-ligand complexes

**SCHEME 1:**
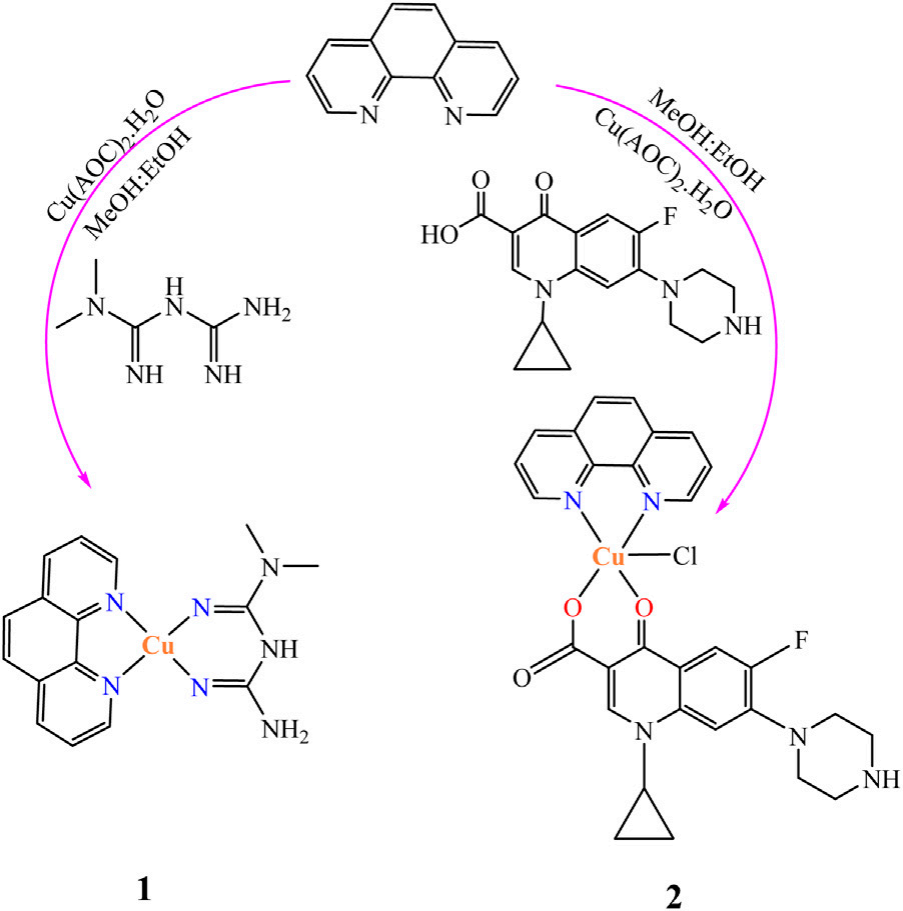
The synthesis procedure of the two Cu(II) complexes (**1** and **2**).

Cu(II) complexes containing metformin and ciprofloxacin as main ligands and 1,10-phenathroline monohydrate as a co-ligand were prepared as reported in the literature (Vasantha et al., 2018) with minor modifications. In a 50 ml round bottom flask equipped with a magnetic stirrer, a methanolic solution of 1,10-phenanthroline monohydrate (0.198 g, 1 mmol) was added to a methanolic solution of Cu(CO_2_CH_3_)_2_·H_2_O (0.166 g, 1 mmol) and stirred for 30 min at room temperature to synthesize the Cu(II) complex (**1**), followed by addition of a hydro-methanolic solution of metformin (0.166 g, 1 mmol; 5:1 methanol-water). On the other hand, a hydro-methanolic solution of ciprofloxacin dissolved in the presence of aqueous NaHCO_3_ (1 mmol) was added slowly from a dropping funnel under continuous stirring to synthesize the second Cu(II) complex (**2**). The design of the synthesized mixed ligand complexes is presented in Scheme 1.

The resulting deep blue solution for complex **1** and green for **2** were refluxed on a hot plate for 3 h each giving brown and green powders, respectively. The reaction progress was monitored using TLC. The obtained precipitates were separated out, filtered off, washed with ice cold methanol and dried over CaCl_2_. The presence of ionizable chloride ion in the ionization sphere of the synthesized metal complexes was checked using 1 M solution of AgNO_3_ test. Negative results for white precipitation confirmed the absence of ionizable chloride ion in the ionization sphere of all the synthesized mixed ligand metal complexes. The complexes are soluble in DMSO and slightly warm water; however, they are insoluble in other organic solvents.

### Instrumentation

The infrared measurements (KBr discs) were recorded on a Perkin-Elmer BX FTIR spectrometer (4000–400). The UV-visible spectra were recorded on a SM-1600 Spectrophotometer. Molar conductance of the complexes was recorded at room temperature in 1.0 × 10-3 M in DMSO solution using electrical conductometer (AD8000). The thermogravimetric/differential thermal analyses (TGA/DTA) data were recorded from 25–800 C with a heating rate of 10°C/min under nitrogen-atmosphere (20 ml/min) by a Shimadzu DTG-60H thermal analyzer. The high-resolution mass spectra were obtained with a Waters-LCT-Premier mass spectrometer using 2 ng/μl of sample concentration with a capillary voltage of 2500 V and a desolvation temperature of 250°C using nitrogen gas at 250 L/h, Bruker APEX II CCD area detector diffractometer, with graphite monochromated Mo K3 radiation (50 kV, 30 mA) and temperature of measurement at 173 (2) K coupled with APEX 2 data collection software. Four scans of width 0.5 and 512 × 512 bit data frames were involved for data collection, SAINT + program was used to achieve the data reduction, and face indexed absorption corrections were made using XPRE.

### Antibacterial activity

The *in vitro* antibacterial activities of the metal complexes were evaluated against the gram-positive bacteria (*E.coli* and *P. aeruginosa*) and gram-negative bacteria (*S. aureus* and *S. pyogenes*) by using disk diffusion method in Mueller Hinton-Agar (MHA) medium. Ciprofloxacin was used as a positive control (Ommenya et al., 2020). Known concentrations (25 and 50 μg/ml) of the test compounds and the positive control were prepared in DMSO. DMSO was used as a negative control and no activity was found. The activity results were presented as a mean of the triplicates.

### Cells and cell maintenance

MCF-7 breast cancer cells previously stored in liquid nitrogen at −180°C, were cultured at 37°C in a humidified 5% CO_2_ atmosphere. The cells were cultured in Dulbecco’s modified eagle medium (DMEM) supplemented with 10% Fetal Bovine Serum (FBS), 2 mM L-glutamine, 50 IU/ml penicillin, and 50 µg/ml streptomycin (Koobotse et al., 2020).

### Cytotoxicity assay

Cytotoxicity of the compounds (**1** and **2**) was evaluated using 3-[4,5-dimethylthiazole-2-yl]-2,5-diphenyltetrazolium bromide (MTT) assay as per manufacturer instructions (Roche). Briefly, MCF-7 breast cancer cells were seeded in a clear, flat-bottom 96-well plate at a density of 5 × 10^3^ cells well^−1^ in 100 µl of growth medium. The cells were allowed to attach for 24 h. The synthesized compounds were dissolved in phosphate-buffered saline (abbreviated PBS, pH ∼ 7.4) and serially diluted with growth media to prepare different concentrations ranging from 25 to 0.78 µM. Spent growth media was discarded and the cells were then treated with the compounds diluted with growth media for 24 h at 37.5°C. Control cells were treated with a mixed solvent (PBS) at concentrations matching those of the compounds following the same procedure. Cisplatin was included as a positive control. After the 24-h incubation period, 10 µl of MTT labelling reagent (final concentration of 0.5 mg/ml) was added to each well and incubated at 37.5°C for 4 h in a humidified 5% CO_2_ atmosphere. DMSO was added to each well to dissolve formazan crystals. Finally, optical density was measured at 570 nm using a Multiskan™ FC Microplate photometer (ThermoFisher Scientific). Three independent experiments were performed.

### Computational

Density functional theory (DFT) calculations employing B3LYP (Lee et al., 1988; Becke, 1993; Stephens et al., 1994) hybrid functional together with 6–311++G (d, p) basis set (Krishnan et al., 1980) for atoms (H, C, N, F, O and Cl) of chelating agents were used to study the quantum chemical properties of the synthesized metal complexes using the Gaussian 16 program package. Los Alamos National Laboratory 2-Double-zeta (LanL2DZ) pseudopotentials were applied for the metal atom [Cu(II)] (Hay and Wadt, 1985) to account for relativistic effects. The non-bonding interactions during the calculations were corrected using Grimme’s dispersion correction (Grimme et al., 2002). This is because such a combination of functional and basis sets has been used and gave results that are in a good agreement with the experiment in our previous studies (Demissie and Hansen, 2016; Demissie et al., 2021). The vibrational frequency calculations were performed at the same level of theory to confirm the optimized geometries were real minima without any imaginary vibrational frequency. The Frontier molecular orbitals (FMOs): highest occupied molecular orbital (HOMO) and lowest unoccupied molecular orbitals (LUMO), and quantum chemical descriptors: energy gap (Δ*E* = *E*_LUMO_ - *E*_HOMO_), electronegativity (*χ* = -½(*E*_HOMO_ + *E*_LUMO_)), electronic chemical potential (*μ* = ½(*E*_HOMO_ + *E*_LUMO_) = -*χ*), global chemical hardness (*η* = ½(_*E*LUMO_ - *E*_HOMO_)), global softness (σ = 1/2η), global electrophilicity index (*ω* = *μ* ^*2*^ */2η*), and nucleophilicity index (*Nu* = 1/*ω*), were calculated and analyzed at the same level of theory (Barakat et al., 2015; Ismael et al., 2020). Such quantum chemical descriptors are pertinent in DFT and are used to establish how the structure, stability and reactivity of compounds relate with their biological activity (Pratiwi et al., 2020).

### Molecular docking studies

Molecular docking studies were performed in order to predict the interaction of the synthesized compounds with the binding sites of estrogen receptor alpha (ERα; PDB:5GS4) (Xie et al., 2017). The geometries of the metal complexes were optimized using the B3LYP-GD3/6-311++G (d,p)/LanL2DZ method prior to docking and converted to PDB files using the Gaussview software. The molecular docking studies of the compounds (**1** and **2**) and the positive control (cisplatin) were performed using the AutoDock 4.2.6 software (Allouche, 2011). The same protocols to our previous works were used (Bitew et al., 2021; Lemilemu et al., 2021; Damena et al., 2022). Briefly, the PDB file for ERα was downloaded from the Protein Data Bank with a resolution of 2.4 Å (Xie et al., 2017). The co-crystallized substrate and water molecules were removed from the receptor using the MGL 1.5.6 software. After cleaning the protein, only polar hydrogens were added together with the Kollman charges. Non-polar hydrogen atoms were merged and Gasteiger partial atomic charges were assigned to the molecules. Standard docking parameters for all the light and metal atoms were used. The grid box was constructed using 120, 120, and 120 pointing in the x, y, and z dimensions, respectively, with a grid point spacing of 0.375 Å. The center grid box was set at −12.055, −10.491, and 5.964 Å for the x, y, and z centers, respectively. Lamarckian genetic algorithm (LGA) program (Morris et al., 1998) with an adaptive whole method search in the AutoDock was selected and set at 100, which generated one hundred different conformations for each of the molecules (Ordog and Grolmusz, 2008). The conformers with the lowest binding free energies were used for the visualization of the interactions between the active amino acids and the molecules using the Discovery Studio software.

### Pharmacokinetic prediction and BOILED egg model

The synthesized metal complexes and the ligands were subjected for physicochemical and pharmacokinetic (ADME) evaluation using SwissADME (Daina et al., 2017). The protocol to predict physicochemical properties, pharmacokinetic and drug likeness evaluation, and BOILED egg model of the metal complexes and the ligands were conducted according to our previous work (Bitew et al., 2021; Lemilemu et al., 2021; Damena et al., 2022).

## Results and discussion

### FTIR analysis

**FIGURE 1:**
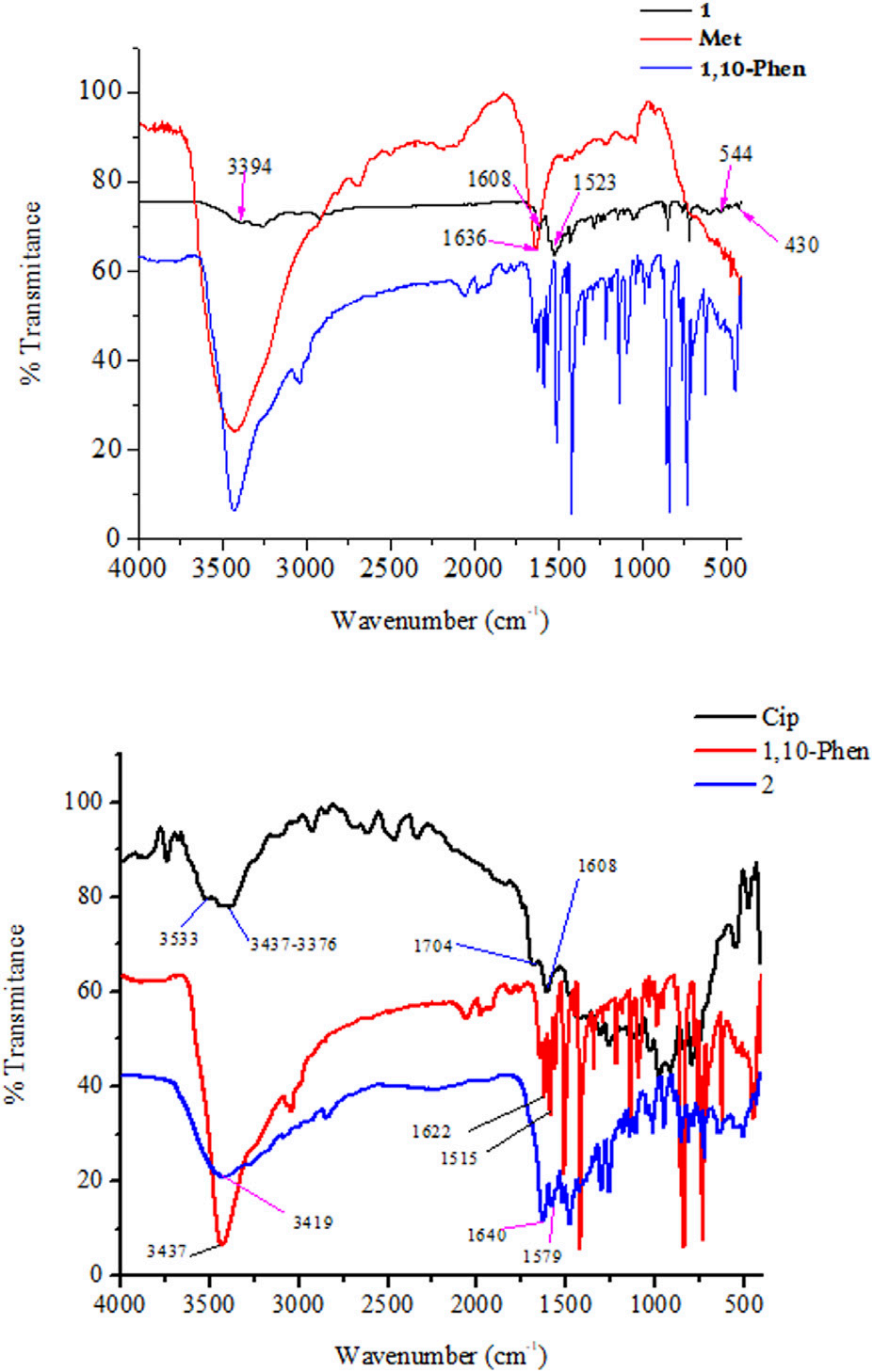
FTIR Spectra of the synthesized metal complexes **1** (top) and **2** (bottom).

The FTIR spectra of the ligands and the synthesized metal complexes are presented in Figure 1 and Table 1; Supplementary Information. It has been reported that ciprofloxacin shows characteristic peaks in the functional group region between 3,500 and 3,450 cm^−1^ for υ(O-H), from 3,000 to 2,950 cm^−1^ for υ(C-H), 1750–1700 cm^−1^ for υ(C=O, carbonyl) and peaks from 1,650 to 1,600 cm^−1^ for υ(C=O, quinolone) (Sahoo et al., 2011). The bands at the 1,450–1,400 cm^−1^ represented υ(C-O) and the peaks at 1,300–1,250 cm^−1^ suggested bending vibration of υ(O-H) group which indicated the presence of carboxylic acid. In addition, a strong absorption peak between 1,050 and 1,000 cm^−1^ was assigned to the C-F group (Patel et al., 2013). Upon coordination to Cu(II) metal center, the characteristic vibrational bands for carboxylic carbon υ(C=O) 1704 cm^−1^ and quinolone carbon (υC = O, 1,608 cm^−1^) shifted to 1,640 cm^−1^ and 1,579 cm^−1^, respectively, confirming the coordination of ciprofloxacin *via* its carboxylic and quinolone oxygen (Figure 1). Moreover, stretching (symmetric and asymmetric) vibrational bands associated to υ(COO)asy and υ(COO) systretches were found to appear at 1,515 cm^−1^ and 1,301 cm^−1^ for ciprofloxacin coordinated Cu(II) complex in the presence of 1,10-phenathroline ligand.

**TABLE 1:** Vibrational frequencies (experimental/calculated) of selected functional groups of the ligands and their metal complexes.

Compound	Selected vibrational frequencies (cm^−1^)							
	ν(OH)	ν(C=O)	ν(N=C)^a^	ν(N-H)	ν(NH_2_)	ν(N=C)^b^	M-O	M-N
Cip	3,520/-	1710/1,694						
1,608/1529^c^	—	3,444/3,406	—	—	—	—		
Met	—	—	1,647/1,643	3,430/3,520	3,423/3,569		—	—
1,10-phen	3,437	—	—	—	—	1,587/1,549	—	—
**1**	—	—	1,626/1,680	3,394/3,510	3,316/3,529	1,523/1,503	—	544/492, 430/416
**2**	—	1,640/1,561	—	3,419/3,442	—	1,615/1,579	509/452	422/423

a b Metformin N=C bond, 1,10-phenathroline N=C bond, c-quinolone C=O.

It has been observed that the FTIR peaks which appeared from 1,615 to 1,430 cm^−1^ in complex **1** was attributed to the coordination of 1,10-phenthroline through its C=N group. In this work, DFT calculation was used to assist the type of metal-nitrogen vibration. Experimental/calculated vibrational frequencies at 544/492 cm^−1^, and 430/416 cm^−1^ in complex **1** are associated to metal Cu-N vibrations of the metformin and 1,10-phenathroline, respectively, confirming the coordination of the ligands (1,10-phenanthroline and metformin) to the metal center Cu(II).

The difference between the asymmetric and symmetric vibrational bands of carboxylic oxygen atoms is useful to determine the coordination mode of ciprofloxacin. In complex **2**, the difference is found to be 214 cm^−1^, inferring the monodentate coordination of COO donor group to the Cu(II) metal center (Patel et al., 2009; Lawal et al., 2020). Whereas, the stretching vibrational bands of 1,10-phenanthroline monohydrate ligand for νC = C (1,622 cm^−1^) and νC = C (1,586 cm^−1^) functional groups are shifted to 1,615 cm^−1^ (w) and 1,515 cm^−1^ (w) with decrease in intensity, respectively. These indicates that the C=C and C=N bond order decreased the ligand coordination to the Cu(II) metal center. On the other hand, the decrease in their intensity indicates the formation of rigid and symmetric structure to the Cu(II) metal center (Abebe et al., 2020). More importantly, the appearance of bands (experimental/calculated) at 509/452 cm^−1^ and 422/423 cm^−1^ (Figure 1) are associated with the formation of Cu-O and Cu-N bonds, respectively.

### Mass spectrometric analysis

The mass spectra of the synthesized metal complexes **1** and **2** are presented in the Supplementary Information. The mass spectrum of **1** shows a molecular ion peak at *m/z* 458.034 (found 459.130) corresponding to [C_16_H_26_CuN_7_O_5_]^+^ (M.Wt of 459.970), a peak at *m/z* 423.064 (423.110) attributed to [C_16_H_22_CuN_7_O_3_] fragment, a *m/z* value of 287.993 (287.040) for [C_13_H_12_CuN_4_] fragment and base peaks at *m/z* values of 181.073 (found 180.210) and 130.103 (found 129.160) corresponding to the constituent ligands, (C_12_H_8_N_2_) and (C_4_H_11_N_5_) fragments, respectively. The mass spectrum of **2** shows a molecular ion peak at *m/z* 612.962 (found 609.540) corresponding to [C_29_H_19_Cl_2_CuFN_5_O_3_]^+^ (M.Wt of 610.120), a peak at *m/z* 573.124 (573.120), is attributed to the two ligands, ciprofloxacin and 1,10-phenathroline, and the metal ion [C_29_H_25_CuFN_5_O_3_]^+^, inferring the proper coordination of the ligands to the Cu(II)-metal center.

### Thermogravimetric analysis

**FIGURE 2:**
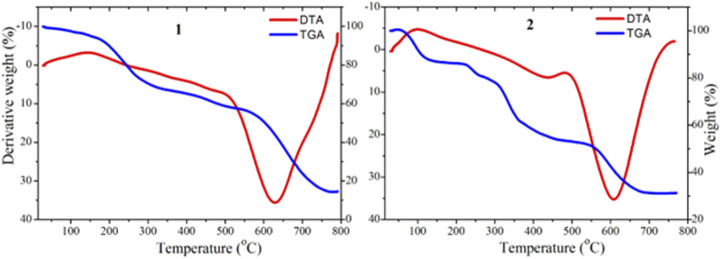
TGA (blue) and DTA (red) curves for complexes **1** and **2**.

The TGA and DTG thermographs assignments of the synthesized complexes are listed in Table 2 and presented in Figure 2. The TGA analysis of complex **1** was performed between 25 and 800°C, and it was found to thermally decompose in four main degradation steps and the thermograph showed that: 1) From 100°C–200°C (DTA_max_ = 180°C), the weight loss was 11.68%, corresponding to the removal of three lattice water molecules, which is in good accordance with the calculated value (11.74%), 2) from 230°C–340°C (DTG max = 300°C), the complex lost its two coordinated water molecules and metformin moiety (2H_2_O+C_4_H_9_N_4_) groups, with the total weight loss being 32.34% (calculated value: 32.41%), 3) from 345°C–570°C, the weight loss is 11.44% (calculated value: 11.52%), corresponding to the decomposition of phenanthroline and release of the C_3_H_3_N organic moiety and the final step showed weight loss 30.42% (calculated value: 30.62%) from temperature 580°C–750°C corresponding to the decomposition of phenanthroline and release of the C_9_H_5_N_2_ organic moiety, and the final residue is related to copper metal with 14.12% (calculated: 13.71%) [2]. The overall weight loss was found to be 85.88% (calculated: 86.29) [1, 2].

**TABLE 2:** Temperature range values for decomposition and corresponding weight loss values.

	Decomposition	Mass loss (%)		Interpretation
	Temp. (^°^C)	Obsd	Calcd	
**1**	100–200	11.68	11.74	loss due to three lattice water molecules
	230–340	32.34	2.41	lost its two coordinated water molecules and metformin moiety (2H_2_O+ C_4_H_9_N_4_) groups
	345–570	11.44	11.52	release of the C_3_H_3_N organic moiety
	580–750	30.42	30.62	release of the C_9_H_5_N_2_ organic moiety
**2**	90–225	13.94	13.96	the release of C_4_H_9_N_2_ molecules
	230–310	8.81	8.93	lost its two chlorine and fluorine molecules moiety (Cl + F)
	320–550	25.22	25.30	release of the C_11_H_8_N organic moiety
	560–700	20.81	20.84	release of the C_9_H_5_N organic moiety

The TGA/DTA analysis of complex **2** (MF = C_29_H_25_ClCuFN_5_O_3_) showed that it thermally decomposed in four main degradation steps and the thermograph showed that: 1) From 90°C–225°C (DTA_max_ = 200°C), the weight loss is 13.94%, corresponding to the release of C_4_H_9_N_2_ fragment, which is in good accordance with the calculated value (13.96%), 2) from 230°C–310°C (DTG max = 270°C), complex **2** lost its chlorine and fluorine moiety (Cl+F) groups, with the total weight loss being 8.81% (calculated value: 8.93%), 3) from 320°C–550°C, the weight loss was 25.22% (calculated value: 25.30%), corresponding to the decomposition of ciprofloxacin and release of the C_11_H_8_N organic moiety and the final step, 4) showed weight loss 20.81% (calculated value: 20.84%) from temperature 560°C–700°C corresponding to the decomposition of phenanthroline and release of the C_9_H_5_N organic moiety. The step total weight loss is 68.78% (calculated: 69.03) with the final residue of related to copper oxide and remaining ligand release (CuO + C_2_H_2_O+ C_3_H_3_N) with 31.22% (calculated: 31.09%) [1, 2].

### UV-vis analysis

**FIGURE 3:**
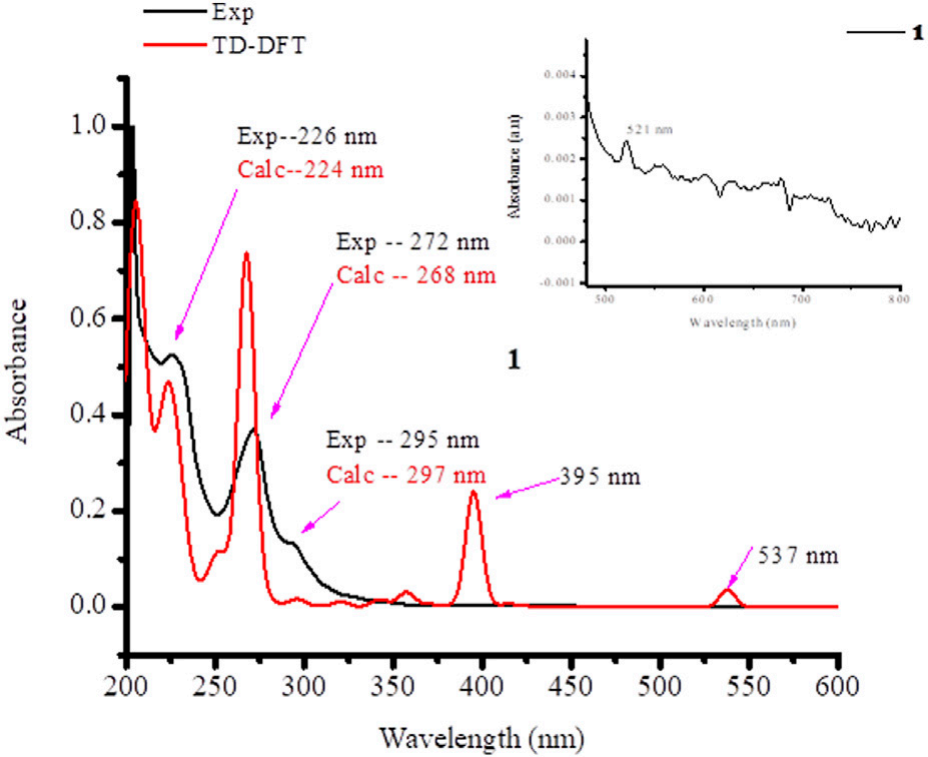
UV-Vis spectra of the synthesized mixed ligand metal complex **1**.

The absorption spectra of the ligands and the synthesized metal complexes (1.0 × 10^-5^ M solution in DMSO) were measured using UV-Vis spectrophotometer. The results obtained from UV-Vis spectrophotometer and TD-DFT calculations are presented in Figure 2; Supplementary Information. The absorption spectra of metformin was observed at 234 nm (Supplementary Information) and in complexes **1** this band has either shifted to 226 nm or appeared as overlapped peaks together with 1,10-phenathroline absorption peaks as shown in the HOMO-LUMO electron distribution (Figure 3). In the UV-Vis spectra of 1,10-Phenathroline free ligand (experimental/TD-DFT) the absorption peaks at 228/226 nm and 263/258 nm corresponds with n→π* (C=N) and π→π*(C=C) transitions, respectively (Tamiru et al., 2019). The peak at 263/258 nm is red shifted to 272/268 nm for the **1** complex. A new peak (experimental/TD-DFT) appeared at 295/297 nm would be associated with the π→π* intraligand transitions between 1,10-phenathroline and metformin electrons, confirming the formation of the required metal nitrogen (M-N) bond. A peak that confirms the presence of charge transfer was appeared at 395 nm in the TD-DFT calculation. The presence of a less intense peak that appeared at 521 nm (Figure 3, shown in inset) was further confirmed by TD-DFT at 537 nm, confirming the presence of 1 d→d electronic transition.

**FIGURE 4:**
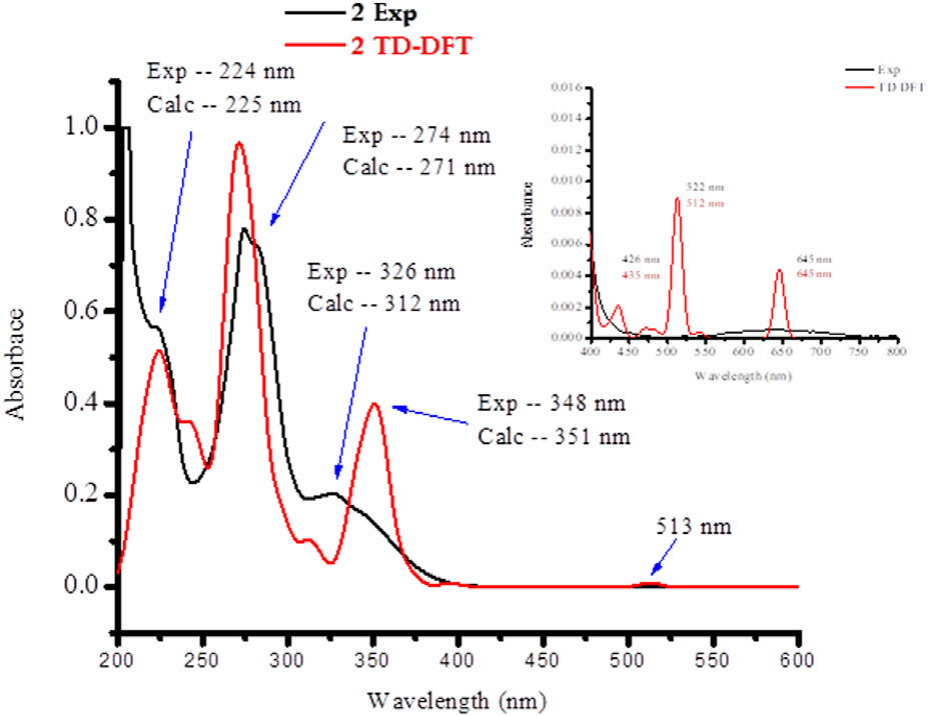
UV-Vis spectra of **2** mixed ligand metal complexes.

In complex **2**, four absorption bands (experimental/TD-DFT) were observed in the UV region of the spectra at 224/225 (n→π*) and 274/271 and 326/312 nm (π→π*) transition of the ligands base peaks, whereas a new peak at 348/351 nm appeared upon coordination to the metal center which could be associated to π→π* electronic transitions due to the formation of metal-oxygen/nitrogen bond (Figure 4). A broad and less intense band observed in the experimental/TD-DFT results that appeared at 645/645 nm could be due to d→d (d_Z_^2^→d_X_^2^_-Y_^2^) (^2^B_1g_ →^2^A_lg_) electronic transitions, confirming the formation of a square pyramidal coordination compound (Hazra et al., 2014).

### Quantum chemical analysis

Analysis of quantum chemical descriptors would be used to relate quantum chemical descriptors of the synthesized metal complexes and their chemical and biological properties. Results obtained from DFT calculations and associated quantum chemical descriptors of the ligands and their metal complexes are presented in Table 3. The HOMO and LUMO *Eigen* values and band gap energy (*E*_g_, *E*_LUMO_—*E*_HOMO_) can be related to the antibacterial, and antioxidant activities. The biological activity of the synthesized complexes towards appropriate molecules can be discussed with the hard–soft–acid–base (HSAB) theory, which states that hard acids prefer to coordinate with hard bases and soft acids with soft bases. Biological molecules such as DNA, enzyme and proteins are soft. Related to this, molecules with small band gap energy are considered soft and those with large band gap energy are hard. There is a direct relationship between biological activity and softness, and inverse relationship with hardness (Ismael et al., 2020; Damena et al., 2022). Consequently, soft complexes can interact easily with biological molecules than the hard ones. Hence, biological activity increases with increasing softness and decreasing hardness. Accordingly, the *E*_g_ was found to be 5.988, 4.755, and 4.096 eV for metformin, 1,10-phenanthroline and ciprofloxacin ligands, respectively. These band gap energy values are relatively decreased upon metal coordination and found to be 2.295 and 2.886 eV, respectively for **1** and **2** (Table 3), inferring the biological importance of the metal complexes than the free ligands. Moreover, results from the band gap energy and dipole moments show that the synthesized metal complexes (**1** and **2)** were found to enhance biological activities. In addition, the calculated electrophilicity (*ω*) and nucleophilicity index (Nu) parameters showed that **1** (5.283 eV) and **2** (5.402 eV) have higher electron acceptor affinity than the constituent ligands. This implies that the synthesized Cu(II) complexes have Lewis acidity (Tandon et al., 2019) than the ligands (Table 3).

**TABLE 3:** Quantum chemical descriptor analysis of the ligands and the synthesized metal complexes.

CPD	HOMO	LUMO	E_g_	Χ	μ	*η*	σ	ω	*Nu*	Dipole Moment
**Met**	−6.440	−0.452	5.988	3.446	−3.446	2.994	0.167	1.983	0.504	5.752
**1,10-Phen**	−6.690	−1.935	4.755	4.313	−4.313	2.377	0.210	3.912	0.256	5.192
**Cipro**	−6.155	−2.059	4.096	4.107	−4.107	2.048	0.244	4.118	0.243	15.128
**1**	−4.630	−2.335	2.295	3.482	−3.482	1.148	0.436	5.283	0.189	9.215
**2**	−6.268	−2.616	2.886	4.442	−4.442	1.826	0.274	5.402	0.185	13.759

**FIGURE 5:**
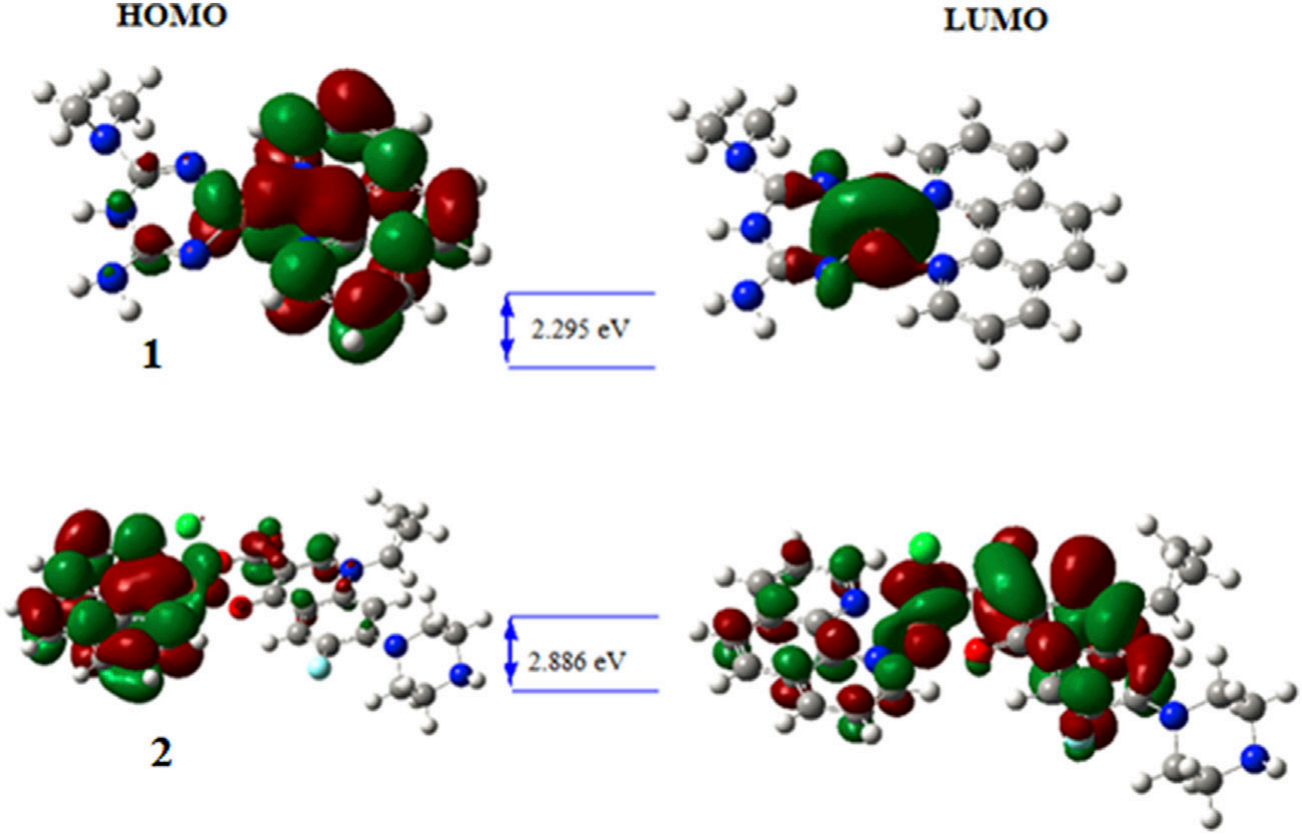
HOMO—LUMO distribution and band gap energy of the synthesized metal complexes.

The wave function distribution of the ligands (metformin, 1,10-phenathroline and ciprofloxacin) and the synthesized metal complexes (**1** and **2**) are in Figure 5; Supplementary Information. The HOMO of the ligands found to reside on dimethyl and quinolone part of metformin and ciprofloxacin, respectively. Equal HOMO-LUMO distributions are observed in 1,10-phenanthroline (supportive information). The HOMO and LUMO distribution of the metal complexes clearly showed the possible electronic tranition. In **1** the HOMO resides on the metal center and 1,10-phenanthroline, confirming the presence of π→π* transition. This distribution found to reside only on the metal center for the LUMO of **1** which confirms the presence of ligand to metal charge transfer (LMCT) and d→d electronic transitions in line with the experimental UV-Vis results.

On the other hand, the HOMO distribution highly resides on the 1,10-phenathroline and metal center, and the LUMO was found to reside on the metal center and ciprofloxacin parts of the molecule in complex **2**, which would be due to π→π* and d→d electronic transitions. In all synthesized metal complexes, 1,10-phenathroline showed to play a significant role in electron transition which would be due to its structural planarity.

### Antibacterial activity of the complexes

**FIGURE 6:**
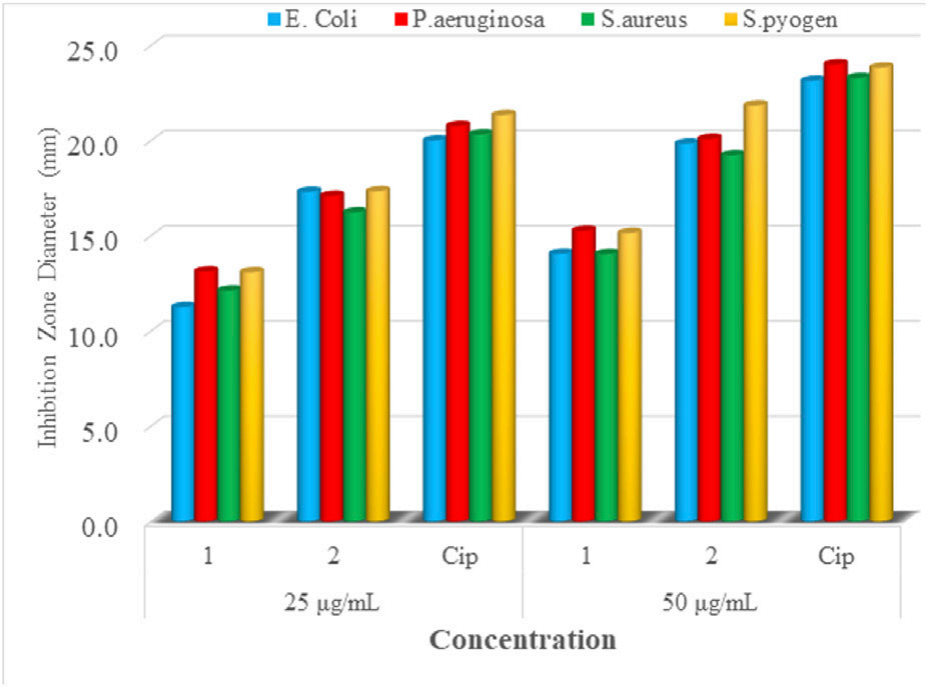
Mean inhibition zone diameter of mixed-drug metal complexes **1** and **2** and ciprofloxacin in mm at 25 and 50 µg/ml.

The synthesized mixed-ligand metal complexes (**1** and **2**) were tested against four bacterial strains (Gram-negative: *E. coli* and *P. aeruginosa*, and Gram-positive: *S. aureus* and *S. pyogen*). The complexes were found to have potent antibacterial activity against both types of bacterial strains. The obtained results are presented in Figure 6; Supplementary Table S2. Among the mixed-ligand Cu(II) complexes, **2** showed strong antibacterial activity at both 25 and 50 μg/ml concentrations against all bacterial strains. This may be due its ligand constituents (1,10-phenathroline and ciprofloxacin) unlike that of **1** which was derived from 1,10-phenathroline and metformin.

### *In vitro* cytotoxicity studies

**FIGURE 7:**
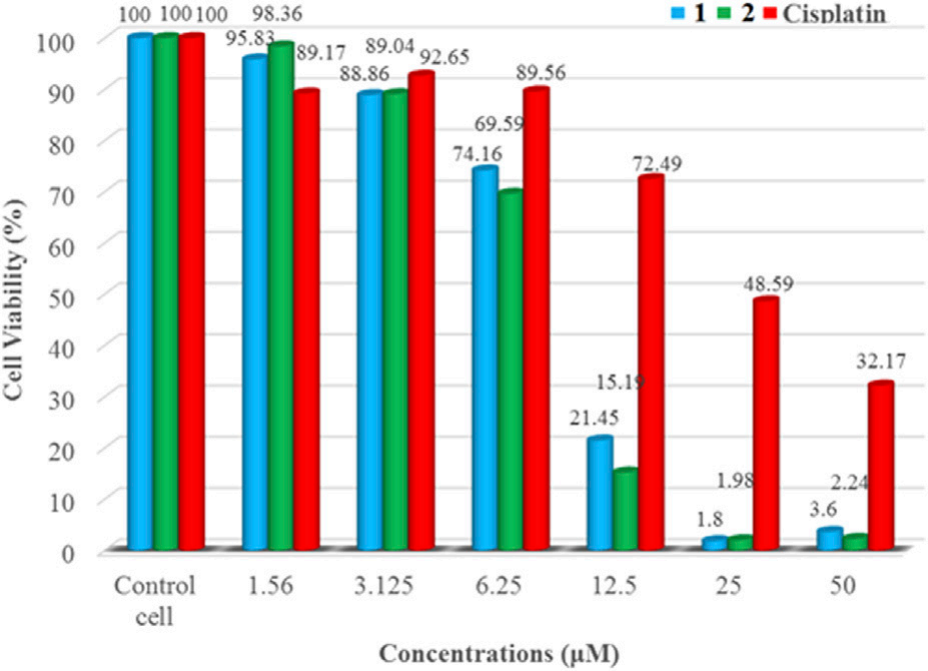
Cell viability of MCF-7 cancer cell line against complexes **1** and **2** and cisplatin.

The *in vitro* cytotoxicity of the synthesized complexes (**1** and **2**) at various concentrations (1.56–25 *µ*M) was evaluated against human breast cancer cell line (MCF-7). The results obtained for cell viability are presented in Figure 7. The Cu(II) complexes showed *in vitro* cytotoxicity against MCF-7 cell line. Increasing concentrations of the synthesized complexes strongly reduced the proliferation of the cancer cells, demonstrating a dose-dependent growth inhibitory effect. Complexes **1** (IC_50_ = 4.29 ± 0.12 µM) and **2** (IC_50_ = 7.58 ± 0.10 µM) were more effective than the referenced chemotherapeutic agent, cisplatin (IC_50_ = 18.62 ± 3.56 µM) (Table 4). Our result are comparable to those reported previously for mixed ligand copper (II) complexes named Casiopeinas® (IC_50_ = 2.2–103.7 *µ*M) against MCF-7 cell line (Bravo-Gomez et al., 2009; Nunes et al., 2020). These could be due to the geometrical and donor atom type resemblance between the metal complexes. The microscopic analysis of the cancer cells (Supplementary Figure S8) treated with complexes **1** and **2** shows a decrease in the number of the cancer cells, confirming the complexes’ ability to induce apoptosis.

**TABLE 4:** *In vitro* cytotoxic activity of complexes **1** and **2** against MCF-7 cell line.

Compound	IC_50_ (*µ*M)
**1**	4.29 ± 0.12
**2**	7.58 ± 0.10
**Cisplatin**	18.62 ± 3.56

### ADMET profile and BOILED egg map evaluation

**FIGURE 8:**
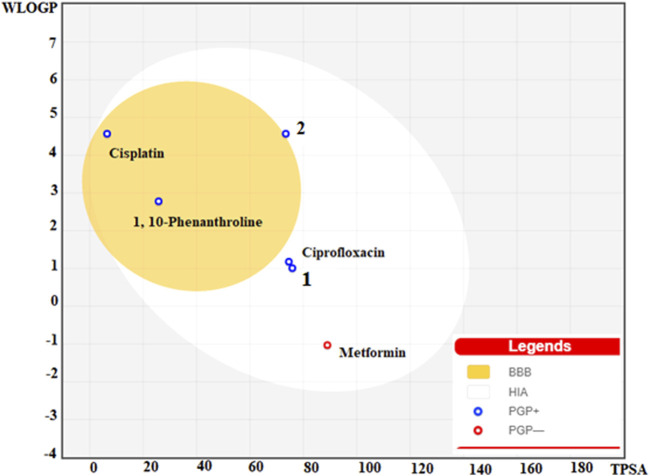
BOILED egg model of the ligands (1,10-Phenanthroline, Ciprofloxacin, and metformin), **1, 2** and Cisplatin.

The physicochemical, drug-likeness and pharmacokinetic prediction profiles of the ligands and their metal complexes are presented in Supplementary Information. A concomitant profiling of the lipophilicity and blood brain barrier permeation are presented in a BOILED egg model (Figure 8). Results obtained from pharmacokinetic profile shows modulation of pharmacokinetic profile of the synthesized metal complexes compared to the constituent ligands (Supplementary Information). This is due to the fact that the metal complexes showed geometrical diversity, high electronegativity difference between the central metal and ligands, and relatively weak bonds between the atoms forming the metal-organic complexes allow the interaction of the desired complexes with biological targets unlike the free ligands (Noffke et al., 2012). The interaction of the ligands and their metal complexes as inhibitors of cytochrome family enzymes: CYP1A2, CYP2C19, CYP2C9, CYP2D6, and CYP3A4 shows that inhibition of CYP1A2 and CYP3A4, and CYP2C19 and CYP3A4 by 1,10-phenathroline and **2**, respectively. Cisplatin was predicted to share similar pharmacokinetic profile with that of complex **2**. It has been reported that the inhibition of CYPs is suggested to induce clinically significant drug-drug interactions (Hakkola et al., 2020), thereby drug induced toxicity. The previously reported toxicity (as neurotoxic, nephrotoxic) (Zhang and Lippard, 2003; Buchtik et al., 2012) of cisplatin would be due to its inhibitory interaction of the CYPs.

Gastrointestinal (GI) absorption and blood-brain barrier (BBB) are vital properties to estimate at various stages of the drug discovery process (Daina and Zoete, 2016). The tendency of the synthesized metal complexes for their GI absorption and BBB permeability was predicted using boiled egg model (Figure 8). The obtained map for the boiled egg model of the complexes is presented in Figure 8. It is found that the lipophilicity and associated neurotoxicity of 1,10-phenathroline has improved upon coordination to a metal center. Our strategy to use the combination of a lipophilic ligand 1,10-phenathroline, and a ligand that has only intestinal absorption resulted in improved metal complexes for their GI absorption, and lipophilicity. Moreover, the synthesized metal complexes showed better GI absorption than that of cisplatin, which is suspected to cause neurotoxicity.

### Molecular docking analysis

**FIGURE 9:**
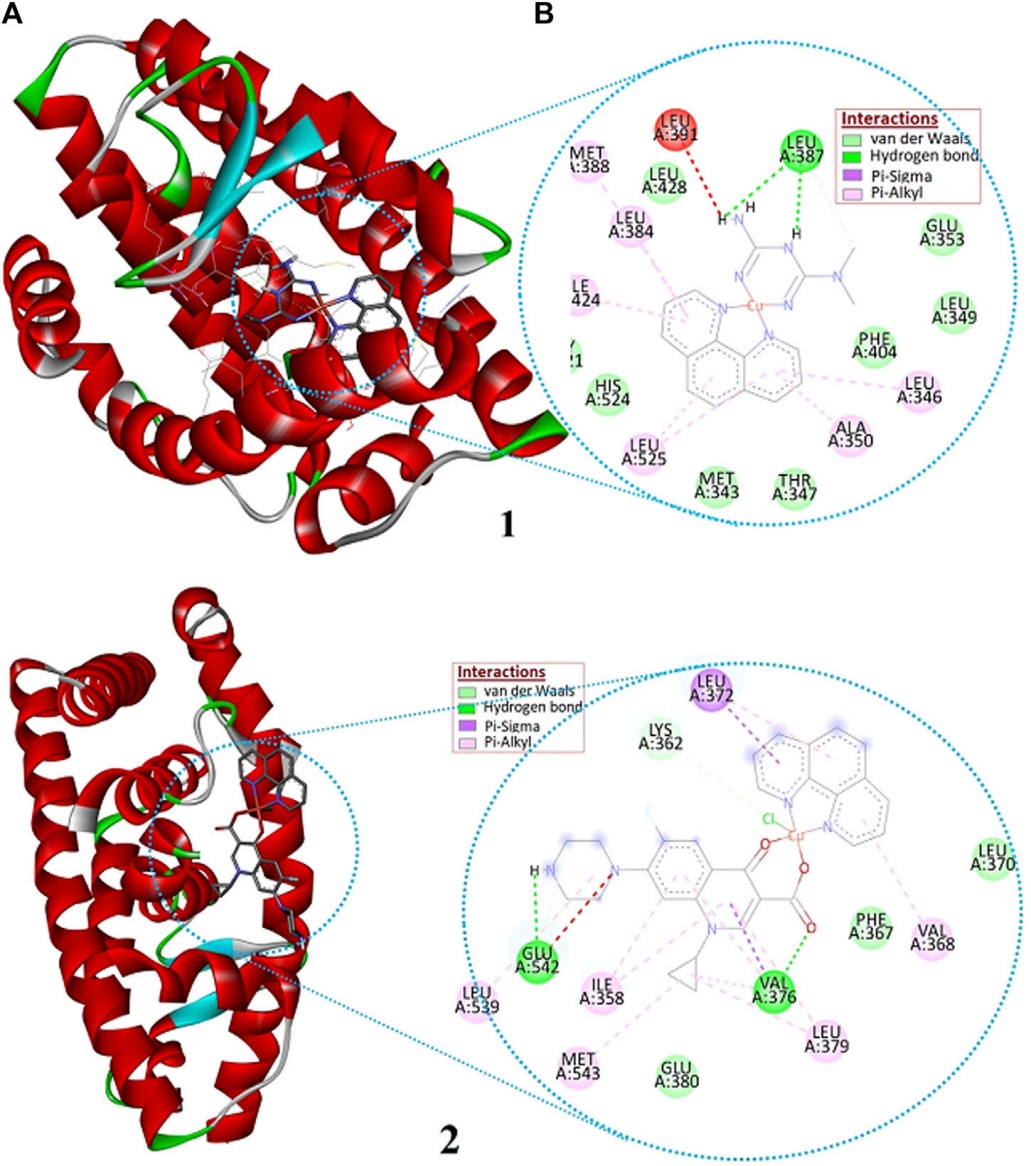
The **(A)** 3D and **(B)** 2D presentations of the binding interactions of complexes **1** and **2** against estrogen receptor alpha (ERα; PDB: 5GS4).

Estrogen receptors (estrogen receptor *α* and estrogen receptor *β*) are the major clinical biomarkers used to subtype breast cancers. The estrogen receptor *α* (ERα) plays an important role in the development and progression of dependent hormonal type breast cancer (Bhatt et al., 2016). The interaction of the synthesized Cu(II) complexes with the prominent residual amino acid interactions of estrogen receptor alpha (ERα; PDB:5GS4) are well presented (Figure 9; Supplementary Information and Table 5). Complex **1** was found to bind to ERα with Leu387 *via* conventional hydrogen bond; Leu525, Ala350 and Ile424 *via* pi-alkyl/pi-sigma interaction; and Phe404, Leu349 and Leu428 *via* van der Waals force with binding affinity of −7.35 kcalmol^-1^. The importance of Arg394 and Glu353 amino acid residues in hydrogen bonding, and Leu387 and Leu391 in pi-alkyl interaction have been reported in a previous study (Pang et al., 2018). The promising anticancer activity of **1** observed in the *in vitro* cytotoxicity study may be associated with a hydrogen bonding interaction with Leu387 amino acid of the active site (Figure 9).

**TABLE 5:** Molecular docking scores and the corresponding prominent residual amino acid interactions of the complexes against estrogen receptor alpha (ERα; PDB:5GS4).

CPDS	Rmsd	Binding energy (Kcal/Mol)	Inhibition constant (*K*_i_)	H (µM)-Bonding with	Π-Sigma/Π-Alkyl	Van der waals
**1**	0.10	−7.35	4.12	Leu387	Leu525, Ala350, Ile424	Phe404, Leu349, Leu428
**2**	0.05	−8.76	0.38	Glu542, Val376	Leu372, Ile358, Leu379	Lys362, Phe367, Glu380
**Cisplatin**	0.39	−6.32	23.42	Ser468, Asp374	Lys467f	Thr371, Glu471

Ile358, Val376, Leu379, Glu380, Leu539, Glu542, and Met543, Phe367, Leu372, Gln375, Val355, Lys362, and Asn359 are amino acids of ERα engaged in hydrophobic interactions. Whereas, Lys362 and Glu542 are proved to be involved in hydrogen bonding (Xie et al., 2017). The higher binding affinity (-8.76 kcalmol^-1^) of **2** would be due to its greater number of interactions with binding site amino acids: two hydrogen bond interaction to Glu542 and Val376, three π-Sigma/π-Alkyl interaction to Leu372, Ile358, and Leu379; and three van der Waals interactions to Lys362, Phe367, and Glu380 (Supplementary Information). The synthesized compounds are found to show any of the three interactions (Table 5) with the binding site amino acids residues of the receptor ERα. However, it is important to note that 1,10-phenathroline based Cu(II) complexes follow multiple mechanism of actions: oxidative damage to DNA, depletion of reduced glutathione, and/or cell death by apoptotic and non-apoptotic dose-dependent DNA binding mechanism (Andiappan et al., 2018).

## Conclusion

In this work, two metal complexes employing antidiabetic and antibacterial drugs as a ligand and 1, 10-phenathroline as a co-ligand were synthesized, and characterized using UV-Vis, FTIR, MS, and TGA/DTA. To shed more-light on the electronic structures of the synthesized metal complexes, computational methods (DFT, TD-DFT and molecular docking analysis) were conducted. The mass to charge ratio (*m/z*) of the synthesized metal complexes were found to be 458.034 and 612.962 for complexes **1** and **2**, respectively, which attributed to molecular formulae [C_16_H_26_CuN_7_O_5_] and [C_29_H_19_Cl_2_CuFN_5_O_3_]. The UV-Vis/TD-DFT and the FTIR/Frequency calculated results are also in very good agreement, confirming the proposed structures. Among the analyzed complexes, complex **2** showed significant antibacterial activity at 25 μg/ml against the two gram-negative (*E. coli* and *P. aeruginosa*) and two gram-positive (*S. aureus* and *S. pyogen*) bacterial strains. More importantly, as per our strategy to test essential metal-based cytotoxic metal therapeutic agents, the complexes were evaluated for their cytotoxic potential against breast cancer cell line (MCF-7). Complexes **1** and **2** were more effective than the referenced drug, cisplatin. Among the two drugs used as ligands, ciprofloxacin showed enhanced antibacterial activity, whereas metformin showed enhanced cytotoxic activity in Cu(II) centered metal complexes. Among the quantum chemical descriptors, the smaller band gap energy (2.295 and 2.886 eV) and higher global softness (0.436 and 0.274 eV) for complexes **1** and **2**, respectively, showed that the metal complexes have biological significance than the bare ligands. It can be concluded that using a square planar Cu(II) complex coordinated to metformin and 1,10-phenathroline would bring promising essential metal-based chemotherapeutic agents. However, we recommend *in-vivo* cytotoxicity study against MCF-7 and MCF-10A cell lines to confirm the promising therapeutic potentials of the complexes.

## Data Availability

The original contributions presented in the study are included in the article/Supplementary Material, further inquiries can be directed to the corresponding authors.

## References

[B1] Abebe A. Bayeh Y. Belay M. Gebretsadik T. Thomas M. Linert W. (2020). Mono and binuclear cobalt (II) mixed ligand complexes containing 1, 10-phenanthroline and adenine using 1, 3-diaminopropane as a spacer: Synthesis, characterization, and antibacterial activity investigations. Future J. Pharm. Sci. 6, 1–9. 10.1186/s43094-020-00030-4

[B2] Allouche A. R. (2011). Gabedit-A graphical user interface for computational chemistry softwares. J. Comput. Chem. 32, 174–182. 10.1002/jcc.21600 20607691

[B3] Andiappan K. Sanmugam A. Deivanayagam E. Karuppasamy K. Kim H.-S. Vikraman D. (2018). *In vitro* cytotoxicity activity of novel Schiff base ligand-lanthanide complexes. Sci. Rep. 8, 1–12. 10.1038/s41598-018-21366-1 29445233 PMC5812993

[B4] Azam A. Raza M. A. Sumrra S. H. (2018). Therapeutic application of zinc and vanadium complexes against diabetes mellitus a coronary disease: A review. Open Chem. 16, 1153–1165. 10.1515/chem-2018-0118

[B5] Barakat A. Soliman S. M. Al-Majid A. M. Lotfy G. Ghabbour H. A. Fun H.-K. (2015). Synthesis and structure investigation of novel pyrimidine-2,4,6-trione derivatives of highly potential biological activity as anti-diabetic agent. J. Mol. Struct. 1098, 365–376. 10.1016/j.molstruc.2015.06.037

[B6] Becke A. D. (1993). Density-functional thermochemistry. III. The role of exact exchange. J. Chem. Phys. 98, 361–363.

[B7] Bhatt S. Stender J. Joshi S. Wu G. Katzenellenbogen B. (2016). OCT-4: A novel estrogen receptor-α collaborator that promotes tamoxifen resistance in breast cancer cells. Oncogene 35, 5722–5734. 10.1038/onc.2016.105 27065334

[B8] Bitew M. Desalegn T. Demissie T. B. Belayneh A. Endale M. Eswaramoorthy R. (2021). Pharmacokinetics and drug-likeness of antidiabetic flavonoids: Molecular docking and DFT study. Plos one 16, e0260853. 10.1371/journal.pone.0260853 34890431 PMC8664201

[B9] Boodram J. N. Mcgregor I. J. Bruno P. M. Cressey P. B. Hemann M. T. Suntharalingam K. (2016). Breast cancer stem cell potent copper(II)-Non-Steroidal anti-inflammatory drug complexes. Angew. Chem. 128, 2895–2900. 10.1002/ange.201510443 26806362

[B10] Bravo-Gómez M. E. García-Ramos J. C. Gracia-Mora I. Ruiz-Azuara L. (2009). Antiproliferative activity and QSAR study of copper (II) mixed chelate [Cu (N–N)(acetylacetonato)] NO3 and [Cu (N–N)(glycinato)] NO3 complexes,(Casiopeínas®). J. Inorg. Biochem. 103, 299–309. 10.1016/j.jinorgbio.2008.10.006 19027166

[B11] Buchtík R. Trávníček Z. Vančo J. (2012). *In vitro* cytotoxicity, DNA cleavage and SOD-mimic activity of copper (II) mixed-ligand quinolinonato complexes. J. Inorg. Biochem. 116, 163–171. 10.1016/j.jinorgbio.2012.07.009 23022693

[B12] Correia I. Roy S. Matos C. P. Borovic S. Butenko N. Cavaco I. (2015). Vanadium(IV) and copper(II) complexes of salicylaldimines and aromatic heterocycles: Cytotoxicity, DNA binding and DNA cleavage properties. J. Inorg. Biochem. 147, 134–146. 10.1016/j.jinorgbio.2015.02.021 25858461

[B13] Daina A. Michielin O. Zoete V. (2017). SwissADME: A free web tool to evaluate pharmacokinetics, drug-likeness and medicinal chemistry friendliness of small molecules. Sci. Rep. 7, 42717. 10.1038/srep42717 28256516 PMC5335600

[B14] Daina A. Zoete V. (2016). A BOILED-egg to predict gastrointestinal absorption and brain penetration of small molecules. ChemMedChem 11, 1117–1121. 10.1002/cmdc.201600182 27218427 PMC5089604

[B15] Damena T. Alem M. B. Zeleke D. Desalegn T. Eswaramoorthy R. Demissie T. B. (2022). Novel zinc (II) and copper (II) complexes of 2-((2-hydroxyethyl) amino) quinoline-3-carbaldehyde for antibacterial and antioxidant activities: A combined experimental, DFT, and docking studies. ACS Omega 30, 26336–26352. 10.1021/acsomega.2c02205 PMC9352163 35936450

[B16] Demissie T. B. Hansen J. H. (2016). Mechanism and site selectivity in visible-light photocatalyzed C-H functionalization: Insights from DFT calculations. J. Org. Chem. 81, 7110–7120. 10.1021/acs.joc.6b00977 27347684

[B17] Demissie T. B. Sundar M. S. Thangavel K. Andrushchenko V. Bedekar A. V. Bouř P. (2021). Origins of optical activity in an oxo-helicene: Experimental and computational studies. ACS omega 6, 2420–2428. 10.1021/acsomega.0c06079 33521480 PMC7841950

[B18] Ferlay J. Colombet M. Soerjomataram I. Parkin D. M. Piñeros M. Znaor A. (2021). Cancer statistics for the year 2020: An overview. Int. J. Cancer 149, 778–789. 10.1002/ijc.33588 33818764

[B19] Grimme S. Furche F. Ahlrichs R. (2002). An improved method for density functional calculations of the frequency-dependent optical rotation. Chem. Phys. Lett. 361, 321–328. 10.1016/s0009-2614(02)00975-2

[B20] Hakkola J. Hukkanen J. Turpeinen M. Pelkonen O. (2020). Inhibition and induction of CYP enzymes in humans: An update. Arch. Toxicol. 94, 3671–3722. 10.1007/s00204-020-02936-7 33111191 PMC7603454

[B21] Hay P. J. Wadt W. R. (1985). *Ab initio* effective core potentials for molecular calculations. Potentials for the transition metal atoms Sc to Hg. J. Chem. Phys. 82, 270–283. 10.1063/1.448799

[B22] Hazra M. Dolai T. Pandey A. Dey S. K. Patra A. (2014). Synthesis and characterisation of copper (II) complexes with tridentate NNO functionalized ligand: Density function theory study, DNA binding mechanism, optical properties, and biological application. Bioinorganic Chem. Appl. 2014, 104046. 10.1155/2014/104046 PMC4214117 25386109

[B23] Ismael M. Abdel-Mawgoud A.-M. M. Rabia M. K. Abdou A. (2020). Design and synthesis of three Fe(III) mixed-ligand complexes: Exploration of their biological and phenoxazinone synthase-like activities. Inorganica Chim. Acta 505, 119443. 10.1016/j.ica.2020.119443

[B24] Johnstone T. Suntharalingam K. Lippard S. (2016). The next generation of platinum drugs: Targeted Pt(II) agents, nanoparticle delivery, and Pt(IV) prodrugs. Chem. Rev. 116, 3436–3486. 10.1021/acs.chemrev.5b00597 26865551 PMC4792284

[B25] Koobotse M. O. Schmidt D. Holly J. M. Perks C. M. (2020). Glucose concentration in cell culture medium influences the BRCA1-mediated regulation of the lipogenic action of IGF-I in breast cancer cells. Ijms 21, 8674. 10.3390/ijms21228674 PMC7698585 33212987

[B26] Krishnan R. Binkley J. S. Seeger R. Pople J. A. (1980). Self‐consistent molecular orbital methods. XX. A basis set for correlated wave functions. J. Chem. Phys. 72, 650–654. 10.1063/1.438955

[B27] Lawal M. Obaleye J. Jadeja R. Bamigboye M. Gupta V. Roy H. (2020). Copper(II) mixed-ligand complexes with fluoroquinolones and an N-donor co-ligand: Structures and biological application. Polyhedron 190, 114753. 10.1016/j.poly.2020.114753

[B28] Lee C. Yang W. Parr R. G. (1988). Development of the Colle-Salvetti correlation-energy formula into a functional of the electron density. Phys. Rev. B 37, 785–789. 10.1103/physrevb.37.785 9944570

[B29] Lemilemu F. Bitew M. Demissie T. B. Eswaramoorthy R. Endale M. (2021). Synthesis, antibacterial and antioxidant activities of thiazole-based schiff base derivatives: A combined experimental and computational study. BMC Chem. 15, 67–18. 10.1186/s13065-021-00791-w 34949213 PMC8697436

[B30] Mitra K. (2016). Platinum complexes as light promoted anticancer agents: A redefined strategy for controlled activation. Dalton Trans. 45, 19157–19171. 10.1039/c6dt03665a 27883129

[B31] Morris G. M. Goodsell D. S. Halliday R. S. Huey R. Hart W. E. Belew R. K. (1998). Automated docking using a Lamarckian genetic algorithm and an empirical binding free energy function. J. Comput. Chem. 19, 1639–1662. 10.1002/(sici)1096-987x(19981115)19:14<1639:aid-jcc10>3.0.co;2-b

[B32] Nishimura Y. Acoba J. D. (2022). Impact of breast cancer awareness month on public interest in the United States between 2012 and 2021: A google trends analysis. Cancers 14, 2534. 10.3390/cancers14102534 35626141 PMC9140129

[B33] Noffke A. L. Habtemariam A. Pizarro A. M. Sadler P. J. (2012). Designing organometallic compounds for catalysis and therapy. Chem. Commun. 48, 5219–5246. 10.1039/c2cc30678f 22517189

[B34] Nunes P. Correia I. Marques F. Matos A. P. dos Santos M. M. Azevedo C. G. (2020). Copper complexes with 1,10-phenanthroline derivatives: Underlying factors affecting their cytotoxicity. Inorg. Chem. 59, 9116–9134. 10.1021/acs.inorgchem.0c00925 32578983

[B35] Ommenya F. Nyawade E. Andala D. Kinyua J. (2020). Synthesis, characterization and antibacterial activity of Schiff base, 4-Chloro-2-{(E)-[(4-fluorophenyl) imino] methyl} phenol metal (II) complexes. J. Chem. 2020, 1745236. 10.1155/2020/1745236

[B36] Ördög R. Grolmusz V. (2008). “Evaluating genetic algorithms in protein-ligand docking,” in Bioinformatics research and applications. ISBRA 2008. Lecture notes in computer science. Editors Măndoiu I. Sunderraman R. Zelikovsky A. (Berlin, Heidelberg: Springer), Vol. 4983. 10.1007/978-3-540-79450-9_37

[B37] Pang X. Fu W. Wang J. Kang D. Xu L. Zhao Y. (2018). Identification of estrogen receptor α antagonists from natural products via *in vitro* and *in silico* approaches. Oxidative Med. Cell. Longev. 2018, 6040149. 10.1155/2018/6040149 PMC5971309 29861831

[B38] Patel M. Chhasatia M. Gandhi D. (2009). DNA-interaction and *in vitro* antimicrobial studies of some mixed-ligand complexes of cobalt(II) with fluoroquinolone antibacterial agent ciprofloxacin and some neutral bidentate ligands. Bioorg. Med. Chem. Lett. 19, 2870–2873. 10.1016/j.bmcl.2009.03.078 19359174

[B39] Patel M. N. Patel C. R. Joshi H. N. (2013). Synthesis, characterization and biological studies of mononuclear copper(II) complexes with ciprofloxacin and N, O donor ligands. Inorg. Chem. Commun. 27, 51–55. 10.1016/j.inoche.2012.10.018

[B40] P. P. B. B. B. A. L. P.V. A. L. (2018). Copper-metformin ternary complexes: Thermal, photochemosensitivity and molecular docking studies. Mater. Sci. Eng. C 90, 621–633. 10.1016/j.msec.2018.04.052 29853132

[B41] Pratiwi R. Ibrahim S. Tjahjono D. H. (2020). Reactivity and stability of metalloporphyrin complex formation: DFT and experimental study. Molecules 25, 4221. 10.3390/molecules25184221 PMC7570457 32942553

[B42] Reina M. Hernández-Ayala L. F. Bravo-Gómez M. E. Gómez V. Ruiz-Azuara L. (2021). Second generation of Casiopeinas: A joint experimental and theoretical study. Inorganica Chim. Acta 517, 120201. 10.1016/j.ica.2020.120201

[B43] Ruiz-Azuara L. E. Bravo-Gomez M. (2010). Copper compounds in cancer chemotherapy. Cmc 17, 3606–3615. 10.2174/092986710793213751 20846116

[B44] Sahoo S. Chakraborti C. K. Mishra S. C. (2011). Qualitative analysis of controlled release ciprofloxacin/carbopol 934 mucoadhesive suspension. J. Adv. Pharm. Tech. Res. 2, 195. 10.4103/2231-4040.85541 PMC3217701 22171318

[B45] Shahidian A. Ghassemi M. Mohammadi J. Hashemi M. (2020). “4-immunotherapy,” in Bio-engineering approaches to cancer diagnosis and treatment. Editors Shahidian A. Ghassemi M. Mohammadi J. Hashemi M. (Amsterdam, Netherlands: Academic Press).

[B46] Stephens P. J. Devlin F. J. Chabalowski C. F. Frisch M. J. (1994). *Ab initio* calculation of vibrational absorption and circular dichroism spectra using density functional force fields. J. Phys. Chem. 98, 11623–11627. 10.1021/j100096a001

[B47] Tamiru G. Abebe A. Abebe M. Liyew M. (2019). Synthesis, structural investigation and biological application of new mono- and binuclear cobalt (II) mixed-ligand complexes containing 1,10-phenanthroline, acetamide and ethylenediamine. Eth J Sci Technol 12, 69–91. 10.4314/ejst.v12i1.4

[B48] Tandon H. Chakraborty T. Suhag V. (2019). A new scale of the electrophilicity index invoking the force concept and its application in computing the internuclear bond distance. J. Struct. Chem. 60, 1725–1734. 10.1134/s0022476619110040

[B49] Xie M. Zhao H. Liu Q. Zhu Y. Yin F. Liang Y. (2017). Structural basis of inhibition of erα-coactivator interaction by high-affinity N-terminus isoaspartic acid tethered helical peptides. J. Med. Chem. 60, 8731–8740. 10.1021/acs.jmedchem.7b00732 29045135

[B50] Zhang C. X. Lippard S. J. (2003). New metal complexes as potential therapeutics. Curr. Opin. Chem. Biol. 7, 481–489. 10.1016/s1367-5931(03)00081-4 12941423

