# Supplementary material for: Cytotoxic mixed-ligand complexes of Cu(II): A combined experimental and computational study
Source: Front Chem. 2022 Sep 29;10:1028957. doi: 10.3389/fchem.2022.1028957 (PMC9557196; doi:10.3389/fchem.2022.1028957)
Supplement: Supplementary file 1 [file DataSheet1.PDF]

Cytotoxic Mixed-Ligand Complexes of Cu(II): A Combined Experimental and Computational Study

Mamaru Bitew Alem,[1,*] Tadewos Damena,[1] Tegene Desalegn,[1,*] Moses Koobotse,[2] Rajalakshmanan Eswaramoorthy,[3] Kennedy J. Ngwira,[4] Japheth O. Ombito,[5] Matshediso Zachariah[2] , Taye B. Demissie,[5,*]

[1]Department of Applied Chemistry, Adama Science and Technology University, P.O.Box 1888, Adama, Ethiopia

[2]School of Allied Health Professions, University of Botswana, Notwane Rd, P/bag UB 0022 Gaborone, Botswana

[3]Department of Biomaterials, Saveetha Dental College and Hospitals, Saveetha Institute of Medical and Technical Sciences, Saveetha University, Chennai-600 077, India

[4]Molecular Sciences Institute, School of Chemistry, University of the Witwatersrand, PO Wits, 2050, Johannesburg, South Africa

[5]Department of Chemistry, University of Botswana, Notwane Rd, P/bag UB 0022 Gaborone, Botswana

Correspondence: mamaru2005@gmail.com (MBA)

tegened@yahoo.com (TD)

ORCID Id: https://orcid.org/0000-0003-0239-8326

demissiet@ub.ac.bw (TBD)

ORCID Id: https://orcid.org/:**0000-0001-8735-4933**

Contents

1. **Physicochemical properties** 3
2. **Characterization of the Ligands and their metal complexes** 4
3. **Pharmacokinetic and Biological Activity of the synthesized complexes** 10
4. **Wavefunction distribution and Molecular docking analysis** 11

1. Physicochemical properties

Table S1. Physicochemical properties of the synthesized mixed drug metal complexes

Compounds	Color	Physical state	Yield (%)	Melting point (^{0}C)	Conductivity ($\Omega^{-1}mol^{-1}cm^{2}$)
**1**	Brown	Powder	0.359 g (78.38)	190 – 194	96 ± 0.340
**2**	Green	Powder	0.486 g (79.80)	Decomposes >206	32 ± 0.047

Dimethyl sulfoxide (DMSO) was a solvent used to test the conductivity of the two complexes (**1** and **2**). The molar conductivity results showed that 96 ± 0.340 and 32 ± 0.047 $\Omega^{-1}mol^{-1}cm^{2}$ for **1** and **2**, respectively, confirming the non-electrolytic nature of the synthesized complexes which would be due to the absence of ions in the ionization sphere of the synthesized metal complexes. The relatively high conductivity of complex **1** could be due to the replacement of anionic ligand, metformin by the solvent DMSO which will create a 1:1 type cation – anion ratio resulting increased conductivity.

2. Characterization of the Ligands and their metal complexes

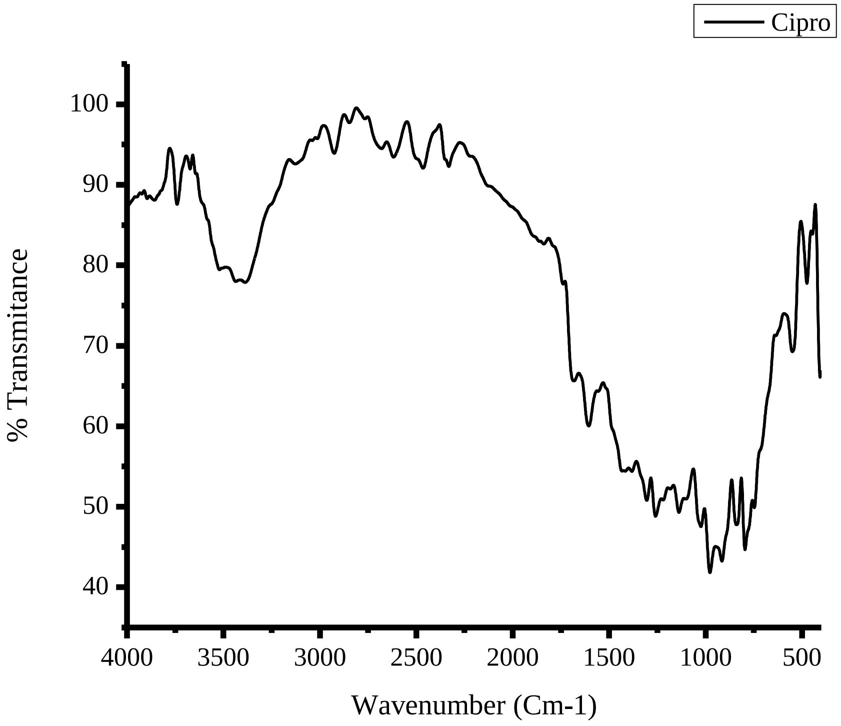

Figure S1. FT-IR Spectra of ciprofloxacin hydrogen chloride (Cip)

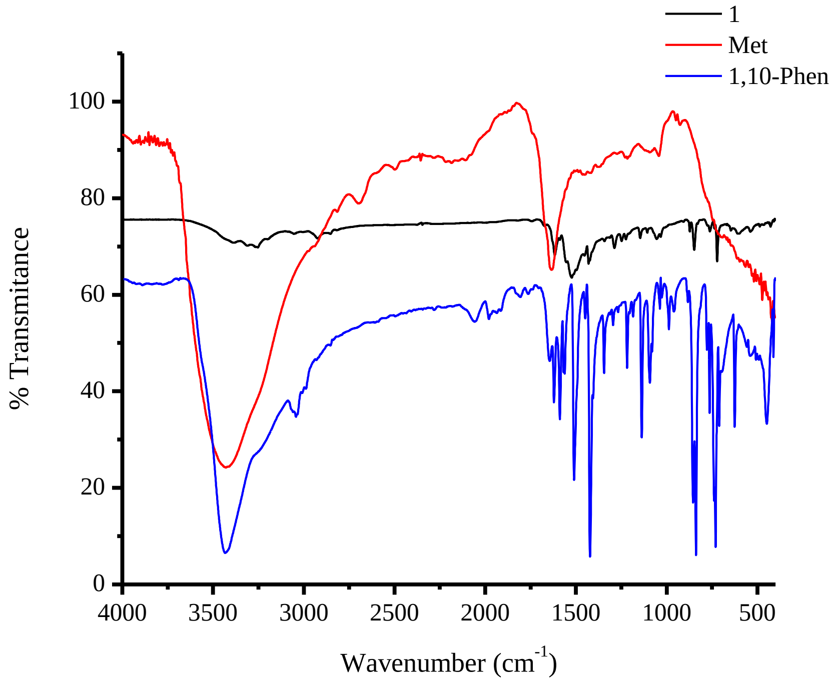

Figure S2. FT-IR Spectra of the synthesized metal complex **2**

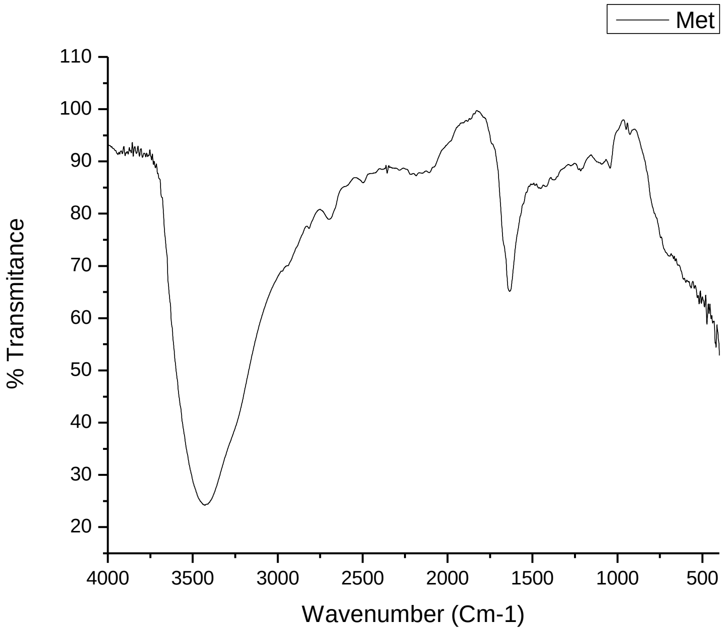

Figure S3. FT-IR Spectra of Metformin hydrogen Chloride (Met. HCl)

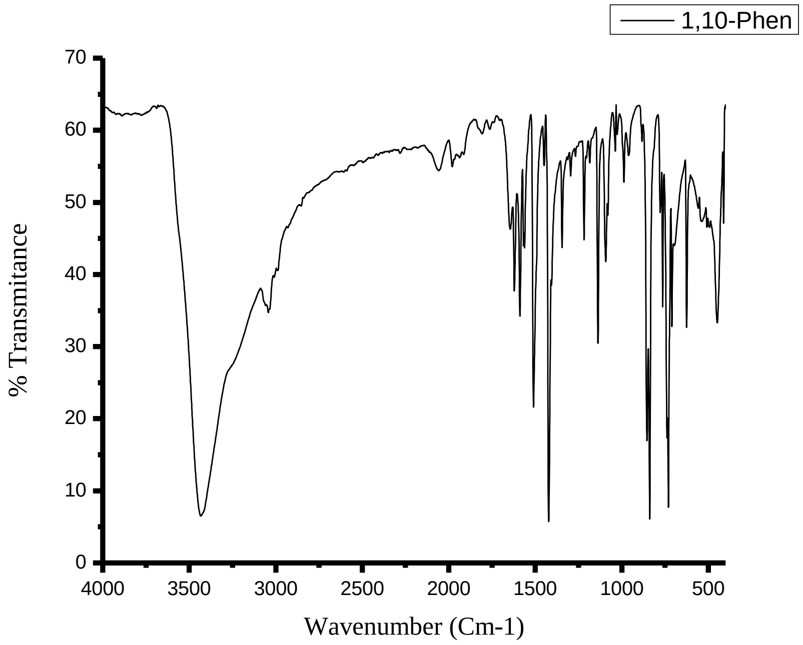

Figure S4. FT-IR Spectra of 1,10-phenanthroline monohydrate

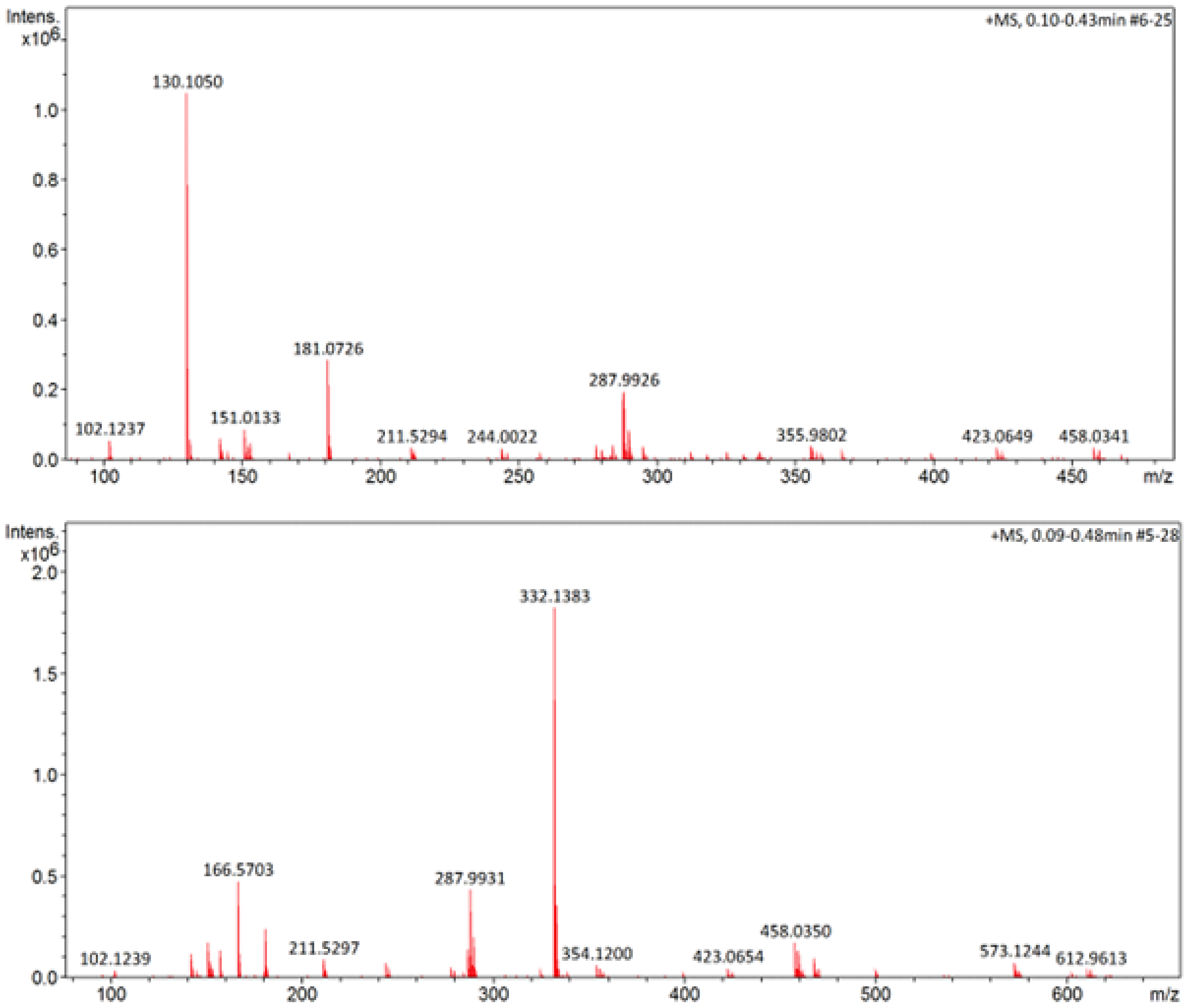

Figure S5. Mass spectrometric analysis of **1** (top) and **2**(bottom)

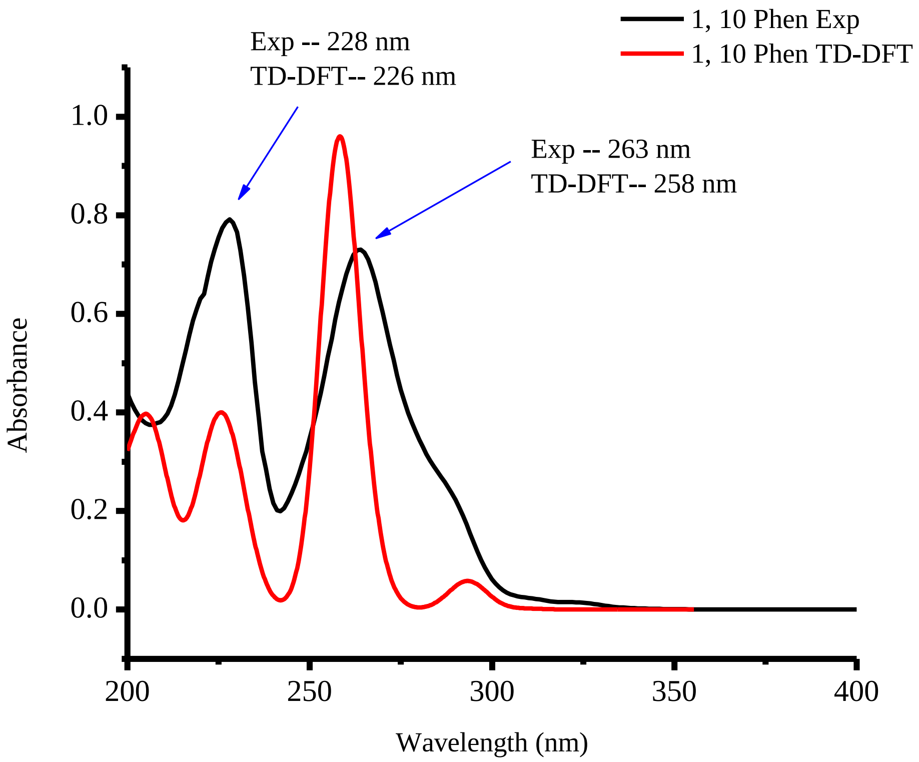

Figure S6. UV-Vis Spectra of 1,10-Phenanthroline monohydrate

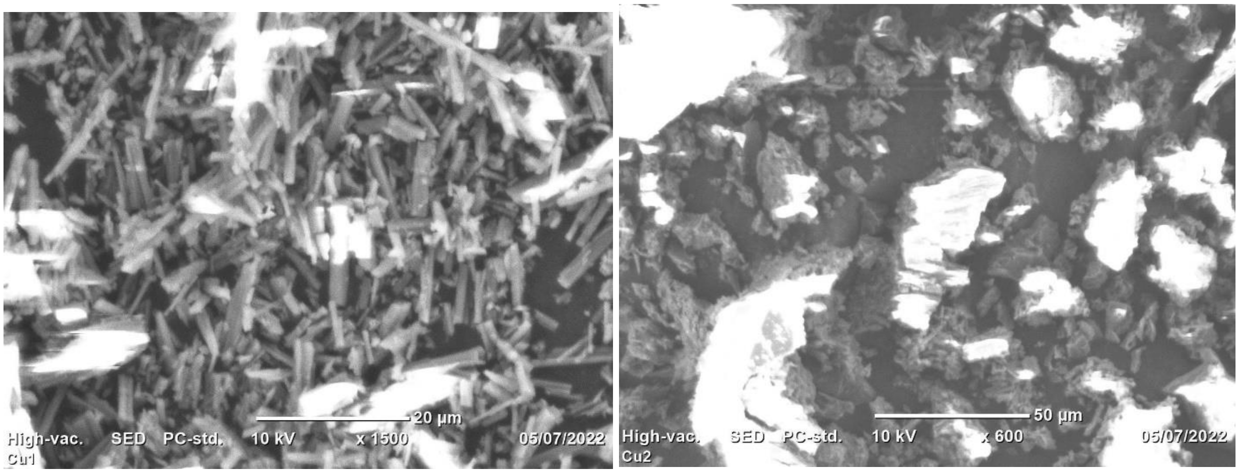

Figure S7. SEM images of the synthesized Cu(II) complexes **1** (left) and **2** (right)

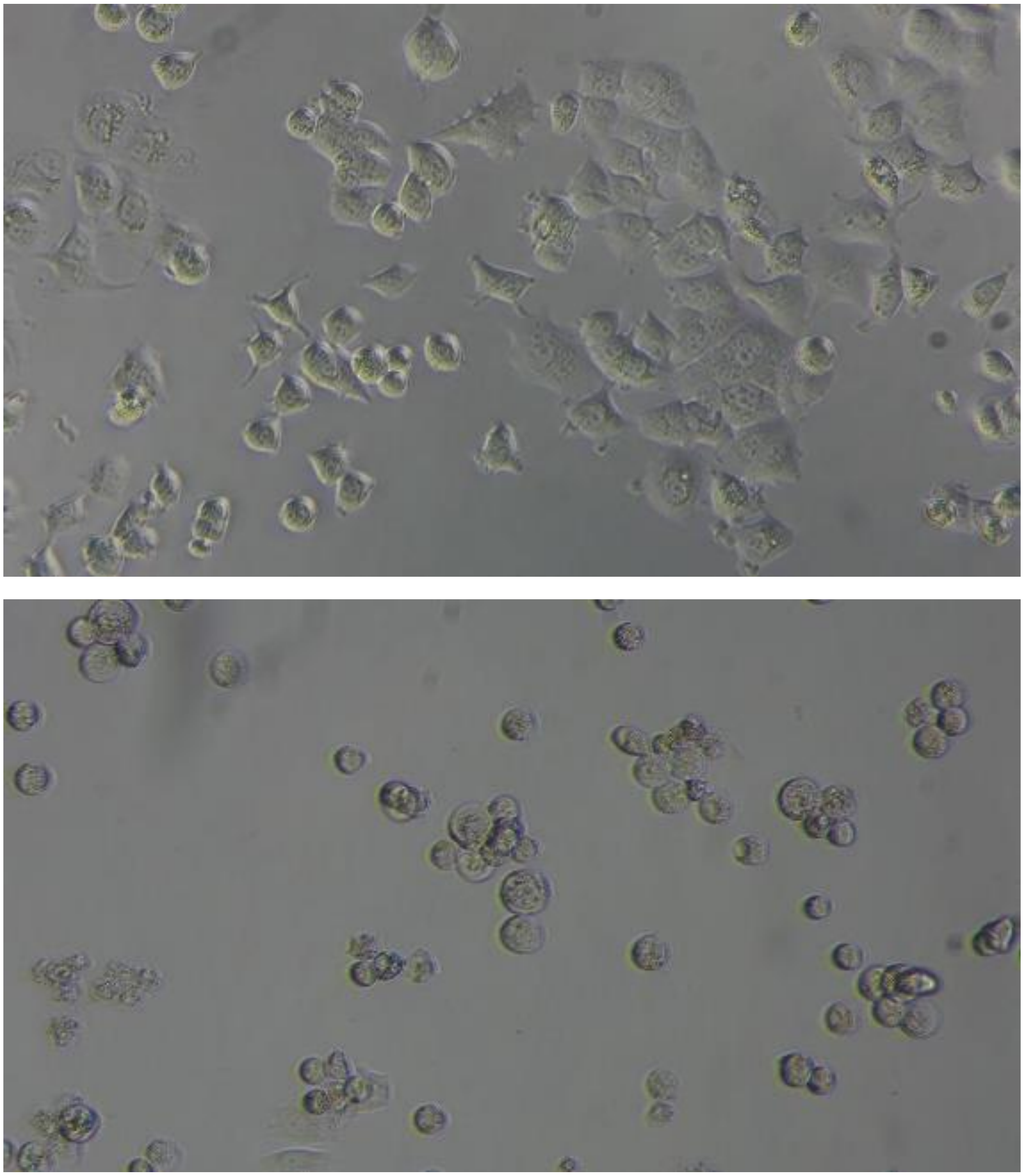

Figure S8. Morphological changes of MCF-7 cell lines induced by: (top) with PBS, and (bottom) with 12.5 μM complex **2**

3. Pharmacokinetic and Biological Activity of the synthesized complexes

Table SI2. Antibacterial activity of complexes (**1** and **2)** and ciprofloxacin

Cpd & Conc.	Bacteria strains and Inhibition Zone Diameter (mm)			
	E. coli ATCC25922	*P. aeruginosa* ATCC27853	*S. aureus* ATCC25923	*S. pyogen* ATCC19615
**1** (25 μg/mL)	11.250 ± 0.204	13.125 ± 0.102	12.117 ± 0.103	13.083 ± 0.312
**2**	17.300 ± 0.216	17.083 ± 0.118	16.217 ± 0.165	17.333 ± 0.471
Cip	20.000 ± 0.354	20.758 ± 0.722	20.317 ± 0.131	21.333 ± 0.471
**1** (50 μg/mL)	14.050 ± 0.268	15.250 ± 0.540	14.033 ± 0.205	15.133 ± 0.189
**2**	19.830 ± 0.118	20.083 ± 0.118	19.217 ± 0.165	21.833 ± 0.624
Cip	23.133 ± 0.189	24.000 ± 0.204	23.283 ± 0.085	23.833 ± 0.312

Table SI3. Physicochemical and drug-likeness screening

Formula	MW	#HBAs	#HBDs	MR	TPSA	iLOGP	Lipinski #violations
$C_{12}H_8N_2$	180.21	2	0	57.04	25.78	1.65	0
$C_4H_{11}N_5$	129.16	2	4	36.93	88.99	0.77	0
$C_{17}H_{18}FN_3O_3$	331.34	5	2	95.25	74.57	2.24	0
$C_{17}H_{21}CuN_7$ (**1**)	386.94	2	2	109.54	75.87	0	0
$C_{29}H_{25}ClCuFN_5O_3$ (**2**)	609.54	5	1	156.6	73.43	0	1
$Cl_2H_6N_2Pt$ (cisplatin)	300.05	5	1	21.16	6.48	0	1

4. Wavefunction distribution and Molecular docking analysis

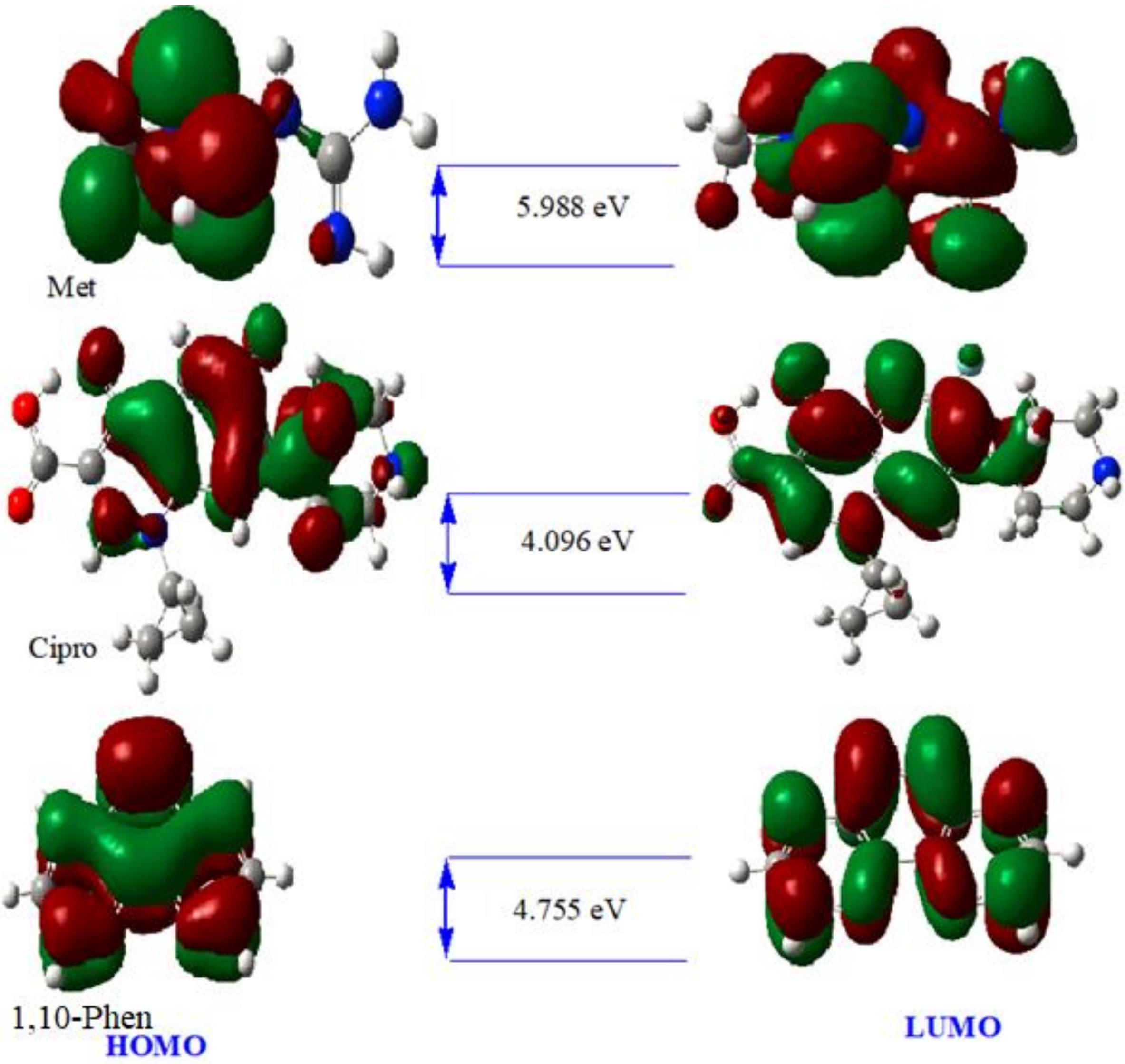

Figure S9. HOMO-LUMO distribution of the lignads

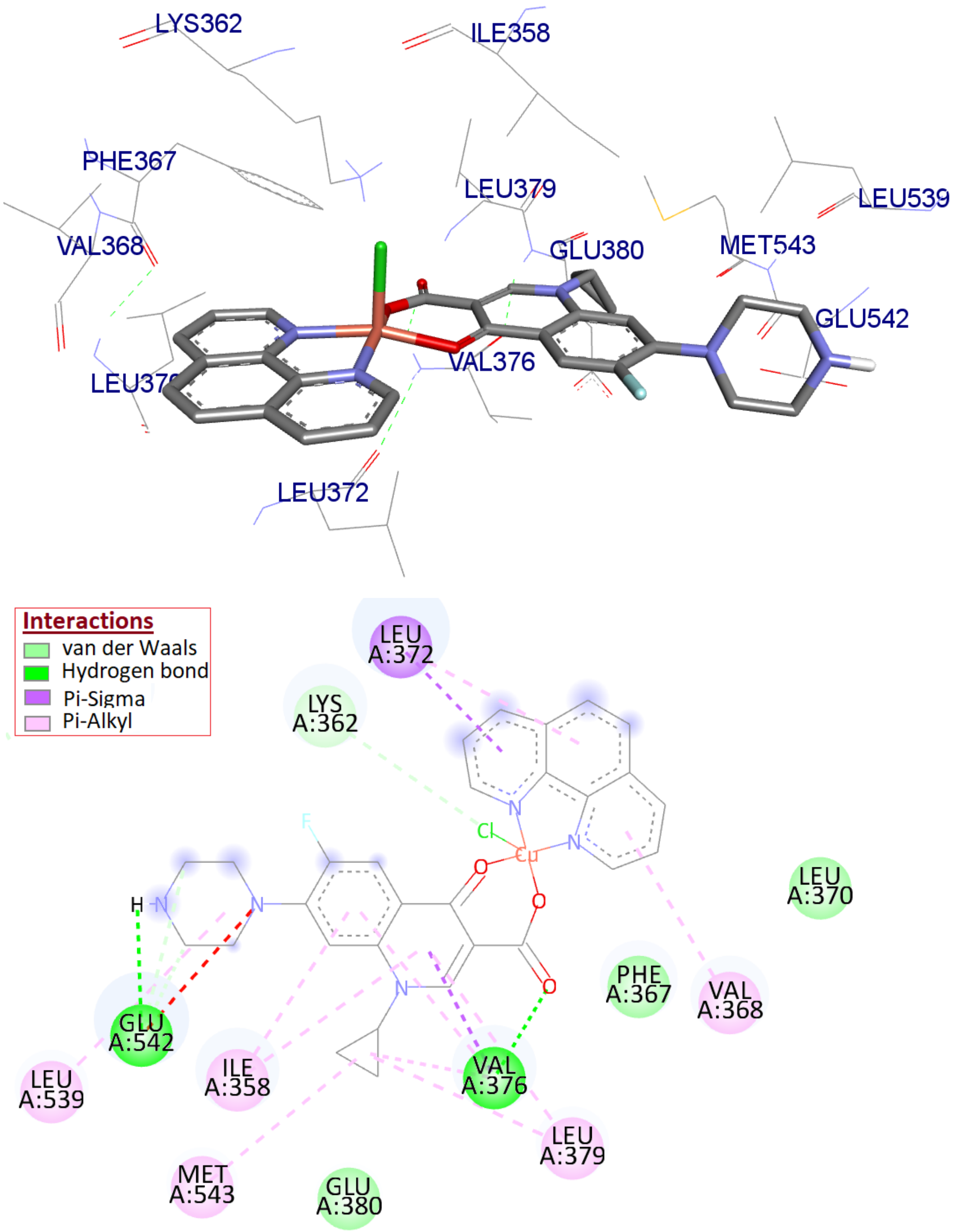

Figure S10. The 3D and 2D binding interactions of complex **2** against estrogen receptor alpha (ERα; PDB:5GS4)
